# Characterising reasons for reversals of COVID-19 vaccination hesitancy among Japanese people: One-year follow-up survey

**DOI:** 10.1016/j.lanwpc.2022.100541

**Published:** 2022-07-21

**Authors:** Shuhei Nomura, Akifumi Eguchi, Daisuke Yoneoka, Michio Murakami, Cyrus Ghaznavi, Stuart Gilmour, Satoshi Kaneko, Takayuki Kawashima, Hiroyuki Kunishima, Wataru Naito, Haruka Sakamoto, Keiko Maruyama-Sakurai, Arata Takahashi, Yoshihiro Takayama, Yuta Tanoue, Yoshiko Yamamoto, Tetsuo Yasutaka, Hiroaki Miyata

**Affiliations:** a Department of Health Policy and Management, School of Medicine, Keio University, Tokyo, Japan; b Department of Global Health Policy, Graduate School of Medicine, The University of Tokyo, Tokyo, Japan; c Tokyo Foundation for Policy Research, Tokyo, Japan; d Center for Preventive Medical Sciences, Chiba University, Chiba, Japan; e Infectious Disease Surveillance Center at the National Institute of Infectious Disease, Tokyo, Japan; f Center for Infectious Disease Education and Research, Osaka University, Osaka, Japan; g Medical Education Program, Washington University School of Medicine in St Louis, Saint Louis, United States; h Graduate School of Public Health, St. Luke's International University, Tokyo, Japan; i Department of Ecoepidemiology, Institute of Tropical Medicine, Nagasaki University, Nagasaki, Japan; j Department of Mathematical and Computing Science, Tokyo Institute of Technology, Tokyo, Japan; k Department of Infectious Diseases, St. Marianna University School of Medicine, Kanagawa, Japan; l Research Institute of Science for Safety and Sustainability, National Institute of Advanced Industrial Science and Technology (AIST), Tsukuba, Ibaraki, Japan; m Department of Hygiene and Public Health, Tokyo Women's Medical University, Tokyo, Japan; n Department of Healthcare Quality Assessment, Graduate School of Medicine, The University of Tokyo, Tokyo, Japan; o Department of International Health and Medical Anthropology, Institute of Tropical Medicine, Nagasaki University, Nagasaki, Japan; p Division of Infectious Diseases, Okinawa Prefectural Chubu Hospital, Uruma, Okinawa, Japan; q Institute for Business and Finance, Waseda University, Tokyo, Japan; r National Center for Child Health and Development, Tokyo, Japan; s Research Institute for Geo-Resources and Environment, National Institute of Advanced Industrial Science and Technology (AIST), Ibaraki, Japan

**Keywords:** Japan, COVID-19, Vaccination hesitancy, Reversals, Reasons

## Abstract

**Background:**

Vaccine hesitancy is a global public health threat. We present unique data that characterises those who experienced reversals of COVID-19 vaccination hesitancy in Japan.

**Methods:**

We administered a questionnaire on vaccination intention among 30053 Japanese adults aged 20 years or older before the COVID-19 vaccination was available to the general population (first survey) and conducted a follow-up survey on vaccination status one year later in February 2022 (second survey). Those who responded in the first survey that they did not intend to be vaccinated or were unsure and then responded in the second survey that they were vaccinated or intend to be vaccinated were asked about the reasons for their change of heart. Based on previous literature and expert opinion, 31 reasons for changing vaccination intention were compiled and respondents were asked to choose which among them applied to themselves, with multiple responses possible. Based on the results of those responses, each individual was then clustered using the Uniform Manifold Approximation and Projection (UMAP) dimensionality reduction technique and Ordering Points To Identify the Clustering Structure (OPTICS) algorithm. We then identified unique characteristics among each of the sub-populations (clusters).

**Findings:**

In the second survey we received 19195 responses (response rate 63.9%), of which 8077 responded ‘no’ or ‘not sure’ in the first survey regarding their intention to be vaccinated. Of these, 5861 responded having received or intending to receive the vaccine (72.6%). We detected six and five sub-populations (clusters) among the ‘no’ group and ‘not sure’ group, respectively. The clusters were characterized by perceived benefits of vaccination, including the COVID-19 vaccine, awareness of the COVID-19 vaccination status of those close to them, recognition of the social significance of COVID-19 vaccination for the spread of infection, and dispelled concerns about short-term adverse reactions and the safety of the COVID-19 vaccine. Work and personal relationship reasons were also found to be a unique overarching reason for vaccination changes of heart only among those who did not intend to vaccinate.

**Interpretation:**

Those who changed their intention to accept COVID-19 vaccination as well as their unique characteristics as detailed in this study will be important entry points when discussing how to promote vaccination to those who are hesitant to vaccinate in the future.

**Funding:**

The present work was supported in part by a grant from the Kanagawa Prefectural Government of Japan and by AIST government subsidies.

**Research in context**

**Evidence before this study**

To explore previous studies with similar scopes, we searched PubMed Central for articles published in English from 1 January 2020 to 29 March 2022 with the following keywords: (“COVID-19”[title] OR “SARS-CoV-2”[title]) AND (“vaccine”[title] OR “vaccines”[title] OR “vaccination”[title]) AND (“intention”[All Fields] OR “hesitant”[All Fields] OR “attitudes”[All Fields] OR “accepting”[All Fields] OR “refusing”[All Fields] OR “resistant”[All Fields]) AND (“follow-up survey”[All Fields] OR “follow-up study”[All Fields] OR “longitudinal study”[All Fields]). Our search yielded 414 titles, but few studies examined changes in intention to receive the COVID-19 vaccine. A few were conducted in the United States and the United Kingdom: for example, a study of people with multiple sclerosis in the United Kingdom and a study of refugees and patients recovering from severe COVID-19 in the United States. In these studies, intent to vaccinate was surveyed before vaccination became available or when the first vaccination campaign was rolled out, and then surveyed again approximately several months to a year later to assess whether there was a change in vaccination intention. Of those who were not willing or unsure to vaccinate, approximately 30% have received the COVID-19 vaccine or made a reservation at follow-up. However, there was only one US-based article that rigorously analysed the reasons for this change in intention, and the reasons included recommendations from physicians, employers, family, and friends, a desire to protect others from the disease, and a sense of security that the vaccine was safe created by knowing someone who received a COVID-19 vaccine and did not get sick afterwards.

**Added value of this study**

This is a one-year follow-up to the survey with a sample size of more than 30,000 people aged 20 years or older in Japan that collected data on their intention to receive COVID-19 before COVID-19 vaccination was initiated in the general population in the country; just under 20,000 responses were received in the follow-up. At the time of follow-up, the third dose of vaccination was being administered in Japan. The follow-up data showed that 46.9% and 80.9% of those who indicated that they did not intend to or were unsure about receiving COVID-19 vaccination have received or showed willingness to receive the vaccine, respectively (72.6% when combined). Based on various literature reviews and in consultation with experts, this study evaluated 31 reasons under the five themes regarding why respondents who indicated that they did not intend to vaccinate or were unsure whether to vaccinate had change of their heart and were vaccinated. Data dimension reduction and clustering techniques, novel approaches in this research area, were used to identify the following unique characteristics of those reasons: the perceived benefits of vaccination, including the COVID-19 vaccine, awareness of the COVID-19 vaccination status of those close to them, recognition of the social significance of COVID-19 vaccination for the spread of infection, dispelled concerns about short-term adverse reactions and the safety of the COVID-19 vaccine, and work and personal relationship reasons.

**Implications of all the available evidence**

As in other countries, achieving the necessary immunization coverage for herd immunity is a major challenge in Japan. This study provides important evidence as to why people in Japan who did not intend to be vaccinated or were unsure have changed their heart and were accepting of the vaccine by follow-up one year later. For example, prior literature encourages vaccination recommendations in the workplace, and our study results support this. If we are to succeed in concerted global vaccination promotion, we need to strengthen our efforts to determine how we can increase vaccine acceptance. This study provides one piece of evidence for more effective public health measures not only by public authorities but also by society as a whole. In addition, because policies and progress on COVID-19 vaccination vary from country to country, not all of the findings of this study in the Japanese context are applicable to other countries or regions, and similar studies are expected in various locations. Indeed, while a study in the United States indicated that recommendations from healthcare professional played an important role in changing the intention to vaccinate against COVID-19, this study found no such finding.

Alt-text: Unlabelled box

## Introduction

COVID-19 has been spreading worldwide for more than two years since the World Health Organization (WHO) declared a global pandemic on March 11, 2020. In the absence of a steady supply of effective treatments, vaccination is one of the most effective public health interventions to prevent and control COVID-19.^1^ Japan's reported cases and deaths from COVID-19 in 2020 were relatively low compared to other high-income countries.^2^ However, since the spring of 2021, COVID-19-related excess deaths have been observed in some areas of the country.^2^ The COVID-19 vaccination campaign began earlier, with priority vaccination of healthcare workers and the elderly in February and April 2021, respectively, and among other populations sequentially thereafter. As of April 10, 2022, 36 COVID-19 vaccines have been approved and 11.39 billion doses have been administered.^3^^,^^4^ In Japan, as of June 1, 2022, four COVID-19 vaccines were approved for use, and 76.0% of the population has completed their two doses of vaccination.^5^

A serious consequence of inadequate COVID-19 vaccine coverage is persistent community transmission (including a surge of highly-contagious variants, such as the Delta and Omicron variants). Low COVID-19 vaccine coverage will further prolong the social and economic impact of the pandemic on families and communities, especially low-income and minority populations.^6^ COVID-19 vaccination campaigns therefore will become increasingly important. Meanwhile, hesitation to COVID-19 vaccines is a major public health challenge. Based on an approach that reflects the ‘attitude roots’ model of science rejection,^7^ many studies have been conducted around the world to identify factors associated with COVID-19 vaccine intention. Their findings suggest that socio-demographic factors such as age, gender, educational level, and socio-economic position are associated with uncertainty and unwillingness to accept a COVID-19 vaccine.^8^^,^^9^ Psychological attributes, including perceived risks of COVID-19, perceived risks and benefits of a COVID-19 vaccine, and trust in governmental institutions have also been associated with being unsure or unwilling to receive COVID-19 vaccines.^10^^,^^11^ Research characterizing why people become vaccine hesitant is essential to create targeted and effective means of disseminating public health messaging regarding COVID-19 vaccine promotion.^11^^,^^12^

Information regarding vaccines and the pandemic situation in general has continued to evolve since the WHO's pandemic declaration, and COVID-19 vaccination intentions may have similarly changed over the year following the start of vaccination programs. In fact, longitudinal studies conducted in the United Kingdom and the United States found that about 30% of those who had expressed that they might not vaccinate against COVID-19 in surveys administered before the vaccines became available had changed their intentions and received vaccinations by the time of follow-up.^13^, ^14^, ^15^ Evidence on the unique, overarching reasons for vaccine hesitancy reversal would provide an important intellectual foundation for promoting vaccination. However, such evidence is scarce and very few studies considered those who were initially vaccine hesitant but became accepting.

At the end of February 2021, before COVID-19 vaccination was available to the general public in Japan, we conducted a COVID-19 vaccination intention survey (hereafter referred to as the first survey) and obtained responses from over 30000 people nationwide. In February 2022, when the COVID-19 booster (third) vaccination campaigns had already begun, we conducted a follow-up survey on the vaccination status (hereafter referred to as the second survey) of the respondents to the first survey; those who changed their intention to be vaccinated from unwilling or unsure to vaccinated or willing to vaccinate were also asked about the reasons for the change. The objective of this study was to characterise the overarching reasons for respondents’ changes of heart using a data dimension reduction and clustering technique. Identification of clusters of people who changed their intention to accept COVID-19 vaccination as well as the unique characteristics of these clusters will be very important entry points when discussing how to promote vaccination to those who are hesitant to vaccinate in the future.

## Methods

### Survey respondents

As described elsewhere, our study participants were recruited from the panel of a web survey company (Cross Marketing Inc.).^10^ The panel consisted of respondents aged 20 years or older who were able to answer the questionnaire in Japanese. Registration on the panel was voluntary. Respondents to the company's surveys are offered "points,” depending on question volume, that can be used to purchase goods and services from partner companies. As of June 2022, this company has over 5 million registered panel members with diverse demographic, socioeconomic, and geographic characteristics.^16^ A total of 30053 people nationwide who responded to the first survey, conducted between 26 February and 4 March 2021, were eligible for the second survey. The subjects of the first survey were recruited according to a quota sampling method, following the population distribution of Japan by age, sex, and prefecture based on the 2015 National Census.^17^

The second survey utilized the same quota sampling method as the first survey, assuming a 70% response rate suggested by the survey company, and began on 4 February 2022 and ended three weeks later on 24 February 2022, with 19195 respondents (response rate 63.9%). Responses to each question were mandatory, so there were no missing data. To avoid dishonest responses, as in the first survey, respondents were asked to take an oath to answer honestly before answering the questionnaire to improve the quality of their survey responses.^18^

### Measures

The questions used in the second survey were roughly the same as those used in the first survey, which was developed based on a thorough review of past literature on vaccination intention and is described in detail elsewhere.^10^ The items in the second survey include the following three parts as in the first survey: health-related topics; psychological characteristics; and sources used to gather information about the COVID-19 pandemic and one's level of trust in each of them. In order to reduce the response burden on the participants, their socio-demographic characteristics were not re-queried in the second survey (except for prefecture of residence), assuming no significant changes since the first survey.

New questions added to the second survey include whether the respondents have received at least one dose of the COVID-19 vaccine. The response options were categorized as ‘vaccinated or scheduled for vaccination’ (*n* = 16291, 84.9%), ‘have not made a reservation but intend to be vaccinated’ (*n* = 254, 1.3%), ‘unsure regarding vaccination’ (*n* = 639, 3.3%), and ‘do not intend to be vaccinated’ (*n* = 2011, 10.5%). For simplicity, the first two categories and the latter two categories are henceforth referred to as 'vaccinated or intending to be vaccinated’ and ‘not sure or not intending to be vaccinated’, respectively. Also, 'vaccinated' in the second survey refers to those who have received at least one vaccination.

Furthermore, those who answered ‘no’ or ‘not sure’ in the first survey regarding their intention to be vaccinated and then indicated that they have received the COVID-19 vaccine or intend to be vaccinated in the second survey (i.e., the study population) were asked about the reasons for their change in intention. The answer choices for the question on why vaccination intention changed were decided based on careful discussion among the authors, who are experts in policy and clinical practice regarding the COVID-19 vaccine in Japan, with reference to the reasons for hesitation to receive COVID-19 identified in previous literature.^8^^,^^9^^,^^19^, ^20^, ^21^, ^22^, ^23^ We established a total of 31 possible reason options that broadly fall under five overarching themes: dispelled specific concerns about the COVID-19 vaccine; change in attitude toward vaccinations in general, not just the COVID-19 vaccine; improved availability of the information necessary to make a decision about COVID-19 vaccination; increased trust in organizations and individuals involved with the COVID-19 vaccine; and others. Other themes included reasons related to health or social conditions relevant to COVID-19, such as to facilitate work or friendships or recommendations from a doctor. The 31 reason texts are shown in the resulting table. Respondents were asked to select the reason(s) that applied to them from a list of 31 possible reasons. The list was structured around the five themes presented above. All 31 options are binary and multiple selections were permitted.

In addition, respondents were asked their opinions on the Japanese government's COVID-19 health pass policy with two questions: ‘Do you agree that various behaviour restrictions should change depending on whether a person has completed COVID-19 vaccination or not (or has a proof of negative COVID-19 test)?’; and for those who do not intend to be vaccinated, ‘Would you vaccinate if various behaviour restrictions change depending on COVID-19 vaccination status (or proof of COVID-19 test results)?’.

As with the first survey, these additional items were developed under the supervision of the Japan Epidemiological Association, the Japanese Society of Infectious Diseases, and experts involved in the COVID-19 Information Value Improvement and Link project (CIVIL project). In this article, only the socio-demographic characteristics of the respondents to the second survey and the study population and their reasons for reversal of vaccine hesitancy are presented in the resulting tables. Socio-demographic data are as of the first survey, except for the prefecture of residence. All questions used in this study are closed-ended and in single- or multiple-response format, and each question item is outlined in the resulting tables. The original Japanese second survey questionnaire and its English translation are included in the supplemental material.

### Study population and statistical analysis

Unless otherwise noted, the study population refers to those who answered ‘no’ (hereafter referred to as Group 1) or ‘not sure’ (hereafter referred to as Group 2) in the first survey regarding their COVID-19 vaccination intention and who indicated that they have received the COVID-19 vaccine or intend to be vaccinated in the second survey.

First socio-demographic data were tabulated for each group as basic information on the study population. Age was considered as a continuous value and the mean age was calculated. Prefecture of residence, gender, occupation, marital status, and presence of underlying disease were treated as categorical or binary variables. Highest education levels and annual household income in 2020 (ordinal scale questions) were also treated as categorical variables. The 19 categories for occupation were reclassified into five new categories: healthcare workers (healthcare and welfare); social and education workers (accommodations, food and beverage services; living-related and personal services and amusement services; education and learning support; students); other essential workers (agriculture, forestry, and fisheries; construction; manufacturing; information and communications; transportation and postal services; wholesale and retail trade; finance and insurance; real estate and good rental and leasing; scientific research, professional, and technical services; public service (not elsewhere classified)); non-essential workers (combines services; services (not elsewhere classified); homemaker); and other (other).

Second, in order to characterise the overarching reasons for respondents’ changes of heart, the data were analysed for Groups 1 and 2, separately, using Uniform Manifold Approximation and Projection (UMAP) and Ordering Points To Identify the Clustering Structure (OPTICS) algorithm. UMAP can identify the data's global structure using dimension reduction techniques (see details in the supplementary material).^24^ In this study, we reduced the 31 binary variables into two-dimensional space for visual inspection. Then, the OPTICS algorithm, which is a recently developed clustering analysis technique, was employed on the reduced dimensional space to identify meaningful clusters of individuals.^25^ Unlike most of the well-known clustering algorithms that require the input of parameter values in advance of the analysis, which are hard to determine but have a significant influence on the results, the OPTICS algorithm requires minimum input of parameter values (e.g., specifying a minimum number of one cluster). In UMAP, a fixed number of nearest neighbours (the default was 15) with hamming distance were used for the binary questionnaire variables. In OPTICS algorithm, the clouds including at least 5% of data points on the two-dimensional space were regarded as a cluster. Threshold values of reachability distances for clustering were set to 2 for Group 1 and 5 for Group 2, respectively. As per the guidelines for the use of OPTICS and elsewhere,^25^^,^^26^ we used simple heuristics to visually determine the threshold values as we only need to ensure the distance values were not too small (Supplementary Figure 1). R version 4.1.2 with packages *uwot* and *dbscan*,^26^ were used for all data analyses.

After clusters were identified and respondents were assigned to their respective clusters, we tabulated the response rates for the 31 reason options for each cluster. We then focused on the difference in the response rates for each reason option across clusters as an indication of the overarching reason characterizing each cluster. We considered a reason option in one cluster that had the highest response rate and differed by more than 5% from the cluster with the second highest response rate to be an important characteristic of that cluster. This 5% value was chosen heuristically; values smaller or larger than 5% did not clearly distinguish the differences in characteristics from other clusters.

Third, the socio-demographic data was tabulated for each cluster for the purpose of comparing differences in socio-demographic characteristics across the clusters.

Finally, we present the results of the above-mentioned two additional questions from the second survey on COVID-19 health pass policy among all respondents to the second survey. The results were separately tabulated for those who indicated in the second survey that they have received or intended to receive the COVID-19 vaccine, and those who indicated in the second survey that they were unsure or did not intend to receive the COVID-19 vaccine.

### Concept of vaccine hesitancy

Vaccination hesitancy is a relatively new concept, and recent research suggests that it is not necessarily reflected by behaviour – or whether or not an individual has actually been vaccinated – but rather that it refers to a psychological state.^27^ Vaccine behaviour is influenced by physical accessibility to vaccination (i.e. convenience^28^) and thus is a health system issue.^27^^,^^29^

For simplicity, the present study defines ‘reversal of vaccine hesitancy’ based on individual attitudes toward COVID-19 vaccination before vaccination started in Japan and actual vaccination behaviour/attitudes after vaccination started. Although this definition does not necessarily align conceptually with that proposed by to Bedford et al.,^27^ we do not consider ‘convenience’ to be relevant to our definition, as the second survey was conducted one year after the start of vaccination.

### Role of the funding source

The funder of the study had no role in study design, data collection, data analysis, data interpretation, or the writing of this report.

## Results

Of the 19195 respondents to the second survey, 1980 (10.3%) responded ‘no’ and 6097 (31.8%) responded ‘not sure’ regarding their intention to be vaccinated against COVID-19 in the first survey. Of each, 928 (46.9%) and 4933 (80.9%) reported having received or intending to receive the COVID-19 vaccine in the second survey, representing Groups 1 and 2, respectively (72.6% when combined); thus, a total of 5861 respondents were analysed in this study. Socio-demographic characteristics of all respondents and the study population are presented in Table 1. Mean age as of the first survey was 54.7 (standard deviation 15.4) and 51.9 (15.2), respectively. While there were more men among the total respondents, there were more women in the study population.

**Table 1:** Socio-demographic characteristics of the respondents.

	Total respondents in the second survey	Study population		
		All	Group 1	Group 2
Number of respondents	19195	5861	928	4933
Age (standard deviation)	54.7 (15.4)	51.9 (15.2)	47.2 (14.9)	52.8 (15.0)
Prefecture of residence (SA)				
Hokkaido	1062 (5.5)	322 (5.5)	52 (5.6)	270 (5.5)
Aomori	155 (0.8)	53 (0.9)	10 (1.1)	43 (0.9)
Iwate	148 (0.8)	51 (0.9)	9 (1.0)	42 (0.9)
Miyagi	376 (2.0)	105 (1.8)	14 (1.5)	91 (1.8)
Akita	122 (0.6)	37 (0.6)	4 (0.4)	33 (0.7)
Yamagata	135 (0.7)	33 (0.6)	5 (0.5)	28 (0.6)
Fukushima	188 (1.0)	57 (1.0)	9 (1.0)	48 (1.0)
Ibaraki	313 (1.6)	93 (1.6)	16 (1.7)	77 (1.6)
Tochigi	171 (0.9)	50 (0.9)	6 (0.6)	44 (0.9)
Gunma	166 (0.9)	46 (0.8)	4 (0.4)	42 (0.9)
Saitama	1066 (5.6)	363 (6.2)	63 (6.8)	300 (6.1)
Chiba	918 (4.8)	272 (4.6)	39 (4.2)	233 (4.7)
Tokyo	2314 (12.1)	683 (11.7)	138 (14.9)	545 (11.0)
Kanagawa	1611 (8.4)	511 (8.7)	63 (6.8)	448 (9.1)
Niigata	372 (1.9)	109 (1.9)	13 (1.4)	96 (1.9)
Toyama	170 (0.9)	49 (0.8)	8 (0.9)	41 (0.8)
Ishikawa	169 (0.9)	44 (0.8)	2 (0.2)	42 (0.9)
Fukui	97 (0.5)	32 (0.5)	3 (0.3)	29 (0.6)
Yamanashi	117 (0.6)	30 (0.5)	6 (0.6)	24 (0.5)
Nagano	322 (1.7)	117 (2.0)	18 (1.9)	99 (2.0)
Gifu	258 (1.3)	82 (1.4)	16 (1.7)	66 (1.3)
Shizuoka	451 (2.3)	148 (2.5)	19 (2.0)	129 (2.6)
Aichi	1276 (6.6)	358 (6.1)	62 (6.7)	296 (6.0)
Mie	263 (1.4)	77 (1.3)	12 (1.3)	65 (1.3)
Shiga	145 (0.8)	40 (0.7)	6 (0.6)	34 (0.7)
Kyoto	396 (2.1)	118 (2.0)	22 (2.4)	96 (1.9)
Osaka	1426 (7.4)	455 (7.8)	59 (6.4)	396 (8.0)
Hyogo	867 (4.5)	271 (4.6)	40 (4.3)	231 (4.7)
Nara	207 (1.1)	66 (1.1)	18 (1.9)	48 (1.0)
Wakayama	82 (0.4)	29 (0.5)	7 (0.8)	22 (0.4)
Tottori	80 (0.4)	25 (0.4)	4 (0.4)	21 (0.4)
Shimane	74 (0.4)	14 (0.2)	1 (0.1)	13 (0.3)
Okayama	332 (1.7)	100 (1.7)	17 (1.8)	83 (1.7)
Hiroshima	464 (2.4)	143 (2.4)	22 (2.4)	121 (2.5)
Yamaguchi	175 (0.9)	53 (0.9)	2 (0.2)	51 (1.0)
Tokushima	116 (0.6)	38 (0.6)	7 (0.8)	31 (0.6)
Kagawa	191 (1.0)	56 (1.0)	9 (1.0)	47 (1.0)
Ehime	231 (1.2)	65 (1.1)	11 (1.2)	54 (1.1)
Kochi	72 (0.4)	20 (0.3)	3 (0.3)	17 (0.3)
Fukuoka	1025 (5.3)	309 (5.3)	53 (5.7)	256 (5.2)
Saga	105 (0.5)	32 (0.5)	2 (0.2)	30 (0.6)
Nagasaki	170 (0.9)	60 (1.0)	9 (1.0)	51 (1.0)
Kumamoto	225 (1.2)	60 (1.0)	12 (1.3)	48 (1.0)
Oita	136 (0.7)	37 (0.6)	7 (0.8)	30 (0.6)
Miyazaki	118 (0.6)	39 (0.7)	5 (0.5)	34 (0.7)
Kagoshima	185 (1.0)	51 (0.9)	14 (1.5)	37 (0.8)
Okinawa	133 (0.7)	58 (1.0)	7 (0.8)	51 (1.0)
Gender (SA)				
Women	9430 (49.1)	3275 (55.9)	476 (51.3)	2799 (56.7)
Men	9744 (50.8)	2582 (44.1)	451 (48.6)	2131 (43.2)
Other	21 (0.1)	4 (0.1)	1 (0.1)	3 (0.1)
Highest educational level (SA)				
Middle school	470 (2.4)	160 (2.7)	19 (2.0)	141 (2.9)
High school	6510 (33.9)	2056 (35.1)	290 (31.2)	1766 (35.8)
Junior college	3643 (19.0)	1222 (20.8)	169 (18.2)	1053 (21.3)
University	7758 (40.4)	2203 (37.6)	398 (42.9)	1805 (36.6)
Graduate school (master's course)	636 (3.3)	179 (3.1)	43 (4.6)	136 (2.8)
Graduate school (doctoral course)	178 (0.9)	41 (0.7)	9 (1.0)	32 (0.6)
Occupation type (SA)				
Healthcare workers	1140 (5.9)	273 (4.7)	58 (6.2)	215 (4.4)
Social and education workers	1572 (8.2)	558 (9.5)	101 (10.9)	457 (9.3)
Other essential workers	6616 (34.5)	2173 (37.1)	374 (40.3)	1799 (36.5)
Non-essential workers	6068 (31.6)	1867 (31.9)	274 (29.5)	1593 (32.3)
Other	3799 (19.8)	990 (16.9)	121 (13.0)	869 (17.6)
Annual household income in 2020 – million JPY (SA)				
–1)	1200 (6.3)	420 (7.2)	69 (7.4)	351 (7.1)
[1–2)	1674 (8.7)	557 (9.5)	97 (10.5)	460 (9.3)
[2–3)	2632 (13.7)	840 (14.3)	124 (13.4)	716 (14.5)
[3–4)	3026 (15.8)	919 (15.7)	127 (13.7)	792 (16.1)
[4–5)	2508 (13.1)	715 (12.2)	109 (11.7)	606 (12.3)
[5–6)	1931 (10.1)	608 (10.4)	87 (9.4)	521 (10.6)
[6–7)	1485 (7.7)	458 (7.8)	74 (8.0)	384 (7.8)
[7–8)	1289 (6.7)	385 (6.6)	74 (8.0)	311 (6.3)
[8–9)	818 (4.3)	236 (4.0)	41 (4.4)	195 (4.0)
[9–10)	805 (4.2)	228 (3.9)	35 (3.8)	193 (3.9)
[10– or	1827 (9.5)	495 (8.4)	91 (9.8)	404 (8.2)
Household size including respondent (SA)				
1	3397 (17.7)	1098 (18.7)	205 (22.1)	893 (18.1)
2	7558 (39.4)	2111 (36.0)	312 (33.6)	1799 (36.5)
3	4500 (23.4)	1409 (24.0)	218 (23.5)	1191 (24.1)
4	2654 (13.8)	906 (15.5)	134 (14.4)	772 (15.6)
5	736 (3.8)	214 (3.7)	38 (4.1)	176 (3.6)
More than 6	350 (1.8)	123 (2.1)	21 (2.3)	102 (2.1)
Marital size (SA)				
Married (including de facto marriage)	12096 (63.0)	3385 (57.8)	483 (52.0)	2902 (58.8)
Not married (without partner)	4216 (22.0)	1528 (26.1)	280 (30.2)	1248 (25.3)
Not married (with a partner)	887 (4.6)	313 (5.3)	68 (7.3)	245 (5.0)
Widowed	751 (3.9)	231 (3.9)	26 (2.8)	205 (4.2)
Divorced	1245 (6.5)	404 (6.9)	71 (7.7)	333 (6.8)
No underlying diseases (SA)	16576 (86.4)	5257 (89.7)	853 (91.9)	4404 (89.3)

Group 1 refers to those who responded in the first survey that they did not intend to vaccinate and subsequently responded in the second survey that they were vaccinated or intending to be vaccinated. Group 2 refers to those who responded in the first survey that they were not sure whether or not to receive the vaccination and subsequently responded in the second survey that they were vaccinated or intending to be vaccinated. SA refers to single-answer questions. Underlying diseases include diabetes, heart failure, respiratory disease, chronic obstructive pulmonary disease, etc., being on dialysis, or using immunosuppressive or anticancer therapies. Data are as of the first survey, except for prefecture of residence.

**Figure 1:**
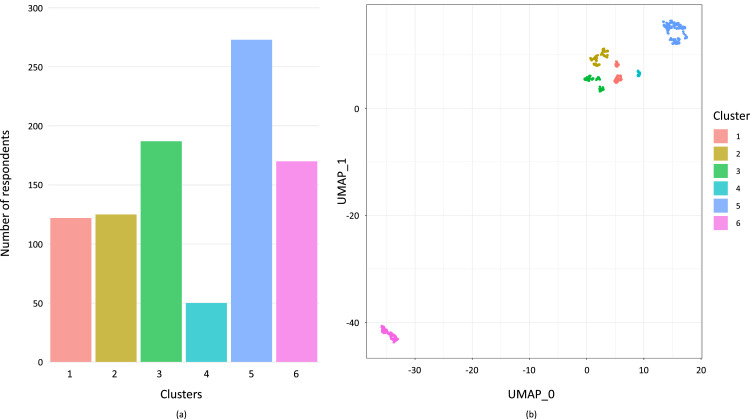
**(a) Distribution of clusters detected by OPTICS on the two-dimensional reduced representation of the data for Group 1; (b) UMAP clusters for two-dimensional reduced representation of the data annotated by the OPTICS generated clusters**.

**Figure 2:**
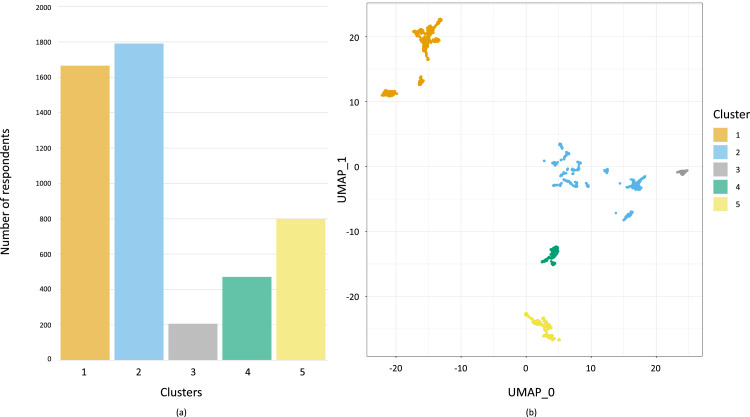
**(a) Distribution of clusters detected by OPTICS on the two-dimensional reduced representation of the data for Group 2; (b) UMAP clusters for two-dimensional reduced representation of the data annotated by the OPTICS generated clusters**.

Using the OPTICS clustering algorithm, we detected six and five sub-populations (clusters) among the study population for Groups 1 and 2, respectively, where one respondent for Group 1 was considered as an outlier. We present the distributions of clusters in Figure 1(a) for Group 1 and in Figure 2(a) for Group 2. Figure 1(b) and Figure 2(b) visualize the Group 1 and Group 2 clusters detected by OPTICS, respectively, by performing UMAP on the two-dimensional reduced representation of our data.

Table 2 presents the cluster-specific responses for the 31 reason options. For Group 1, we refer to the six clusters as follows: Cluster 1 (*n* = 122, 13.2%) that still has had concerns about the effectiveness of the COVID-19 vaccine but has begun to recognize the benefits of vaccination in general; Cluster 2 (*n* = 125, 13.5%) where short-term concerns about the COVID-19 vaccine have been dispelled by someone close to them who received the COVID-19 vaccine; Cluster 3 (*n* = 187, 20.2%) with no clear overarching reason; Cluster 4 (*n* = 50, 5.4%) that considers the COVID-19 vaccine necessary for work and personal relationships; Cluster 5 (*n* = 273, 29.4%) that has begun to recognise the benefits of the COVID-19 vaccine and its social significance with respect to controlling the spread of infection while concerns about short-term and future adverse reactions remain; and Cluster 6 (*n* = 170, 18.3%), for which concerns about short-term adverse reactions and the safety of COVID-19 vaccine have been dispelled to some extent.

**Table 2:** Cluster specific response for all answer options.

	Group 1						Group 2				
	Cluster 1	Cluster 2	Cluster 3	Cluster 4	Cluster 5	Cluster 6	Cluster 1	Cluster 2	Cluster 3	Cluster 4	Cluster 5
Number of respondents (% in row)	122 (13.2)	125 (13.5)	187 (20.2)	50 (5.4)	273 (29.4)	170 (18.3)	1666 (33.8)	1791 (36.3)	206 (4.2)	471 (9.5)	799 (16.2)
Reason options											
Dispelled specific concerns about the COVID-19 vaccine											
1. Concerns about short-term adverse reactions and safety have been dispelled to some extent.	0 (0.0)	0 (0.0)	0 (0.0)	0 (0.0)	12 (4.4)	170 (100.0)*	351 (21.1)	7 (0.4)	0 (0.0)	4 (0.8)	748 (93.6)*
2. Benefits of vaccination outweigh concerns about short-term adverse reactions and safety.	6 (4.9)	2 (1.6)	0 (0.0)	2 (4.0)	176 (64.5)*	21 (12.4)	1655 (99.3)*	0 (0.0)	0 (0.0)	1 (0.2)	3 (0.4)
3. Concerns about future adverse reactions and safety have been dispelled to some extent.	6 (4.9)	48 (38.4)*	2 (1.1)	0 (0.0)	11 (4.0)	13 (7.6)	234 (14.0)	26 (1.5)	204 (99.0)*	7 (1.5)	60 (7.5)
4. Benefits of vaccination outweigh concerns about future adverse reactions and safety.	9 (7.4)	4 (3.2)	1 (0.5)	8 (16.0)	104 (38.1)*	4 (2.4)	480 (28.8)	446 (24.9)	1 (0.5)	10 (2.1)	27 (3.4)
5. Concerns about effectiveness have been dispelled to some extent.	9 (7.4)	41 (32.8)*	2 (1.1)	1 (2.0)	11 (4.0)	8 (4.7)	187 (11.2)	203 (11.3)	5 (2.4)	19 (4.0)	47 (5.9)
6. Benefits of vaccination outweigh concerns about effectiveness.	81 (66.4)*	1 (0.8)	2 (1.1)	3 (6.0)	23 (8.4)	7 (4.1)	324 (19.4)	27 (1.5)	4 (1.9)	468 (99.4)*	26 (3.3)
Change in attitude toward vaccination in general, not just the COVID-19 vaccine											
7. I have begun to believe that vaccines are necessary.	52 (42.6)*	2 (1.6)	4 (2.1)	2 (4.0)	42 (15.4)	40 (23.5)	414 (24.8)	219 (12.2)	24 (11.7)	73 (15.5)	286 (35.8)*
8. I have begun to believe that vaccines are effective.	35 (28.7)*	22 (17.6)	21 (11.2)	0 (0.0)	67 (24.5)	31 (18.2)	688 (41.3)*	313 (17.5)	67 (32.5)	119 (25.3)	259 (32.4)
9. Concerns about adverse reactions and the safety of vaccines have been dispelled to some extent.	12 (9.8)	23 (18.4)	3 (1.6)	0 (0.0)	53 (19.4)	39 (22.9)	395 (23.7)	190 (10.6)	65 (31.6)*	61 (13.0)	153 (19.1)
10. Fear of vaccines has been dispelled to some extent.	11 (9.0)	18 (14.4)	6 (3.2)	1 (2.0)	40 (14.7)	17 (10.0)	243 (14.6)	124 (6.9)	24 (11.7)	94 (20.0)*	66 (8.3)
Availability of the information necessary to make a decision about COVID-19 vaccination											
11. There is now more information about the compatibility of vaccines with certain pre-existing health conditions (e.g., allergies).	25 (20.5)	26 (20.8)	15 (8.0)	2 (4.0)	70 (25.6)	46 (27.1)	619 (37.2)	297 (16.6)	73 (35.4)	137 (29.1)	325 (40.7)
12. There is now more information regarding whether or not someone close to oneself has received the vaccine.	36 (29.5)	73 (58.4)*	5 (2.7)	1 (2.0)	84 (30.8)	41 (24.1)	512 (30.7)	377 (21.0)	82 (39.8)*	142 (30.1)	203 (25.4)
Increased trust in organizations and individuals involved with the COVID-19 vaccine											
13. Trust in public institutions and politicians has increased.	19 (15.6)	17 (13.6)	3 (1.6)	0 (0.0)	26 (9.5)	19 (11.2)	268 (16.1)	94 (5.2)	40 (19.4)	56 (11.9)	151 (18.9)
14. Trust in scientists has increased.	6 (4.9)	9 (7.2)	2 (1.1)	1 (2.0)	27 (9.9)	9 (5.3)	186 (11.2)	50 (2.8)	18 (8.7)	28 (5.9)	56 (7.0)
15. Trust in pharmaceutical companies has increased.	10 (8.2)	17 (13.6)	2 (1.1)	1 (2.0)	34 (12.5)	14 (8.2)	237 (14.2)	87 (4.9)	29 (14.1)	42 (8.9)	75 (9.4)
16. Trust in healthcare professionals has increased.	23 (18.9)	26 (20.8)	3 (1.6)	0 (0.0)	68 (24.9)	30 (17.6)	524 (31.5)	247 (13.8)	75 (36.4)	153 (32.5)	218 (27.3)
Others											
17. A family member has been infected with COVID-19.	2 (1.6)	0 (0.0)	0 (0.0)	0 (0.0)	4 (1.5)	4 (2.4)	24 (1.4)	23 (1.3)	6 (2.9)	6 (1.3)	25 (3.1)
18. A friend or colleague at work has been infected with COVID-19.	1 (0.8)	5 (4.0)	7 (3.7)	1 (2.0)	9 (3.3)	5 (2.9)	54 (3.2)	42 (2.3)	9 (4.4)	13 (2.8)	21 (2.6)
19. A family member has received COVID-19 vaccination.	13 (10.7)	25 (20.0)	31 (16.6)	3 (6.0)	44 (16.1)	19 (11.2)	341 (20.5)	328 (18.3)	25 (12.1)	61 (13.0)	138 (17.3)
20. A friend has received COVID-19 vaccination.	8 (6.6)	21 (16.8)	13 (7.0)	5 (10.0)	19 (7.0)	21 (12.4)	209 (12.5)	185 (10.3)	14 (6.8)	44 (9.3)	65 (8.1)
21. A colleague at work has received COVID-19 vaccination.	5 (4.1)	18 (14.4)	19 (10.2)	7 (14.0)	18 (6.6)	14 (8.2)	128 (7.7)	144 (8.0)	10 (4.9)	28 (5.9)	40 (5.0)
22. My doctor or family doctor recommended COVID-19 vaccination.	5 (4.1)	5 (4.0)	6 (3.2)	0 (0.0)	19 (7.0)	4 (2.4)	148 (8.9)	90 (5.0)	12 (5.8)	26 (5.5)	57 (7.1)
23. My work and personal relationships would improve if I received the COVID-19 vaccination.	4 (3.3)	11 (8.8)	2 (1.1)	50 (100.0)*	22 (8.1)	16 (9.4)	225 (13.5)	216 (12.1)	15 (7.3)	46 (9.8)	51 (6.4)
24. I have been influenced by a TV personality or celebrity.	2 (1.6)	1 (0.8)	3 (1.6)	0 (0.0)	4 (1.5)	1 (0.6)	27 (1.6)	11 (0.6)	3 (1.5)	2 (0.4)	15 (1.9)
25. There is a vaccination site or medical facility near my home where I can receive the vaccine.	5 (4.1)	6 (4.8)	11 (5.9)	2 (4.0)	27 (9.9)	9 (5.3)	344 (20.6)*	156 (8.7)	28 (13.6)	44 (9.3)	86 (10.8)
26. I was concerned about airborne transmission of COVID-19.	7 (5.7)	6 (4.8)	11 (5.9)	2 (4.0)	39 (14.3)	16 (9.4)	357 (21.4)*	211 (11.8)	30 (14.6)	53 (11.3)	103 (12.9)
27. I was worried about the diminishing effect of the emergency declarations and other COVID-19 measures to prevent the spread of the virus.	3 (2.5)	1 (0.8)	3 (1.6)	0 (0.0)	22 (8.1)	9 (5.3)	207 (12.4)*	99 (5.5)	12 (5.8)	24 (5.1)	44 (5.5)
28. I worried about the overwhelmed healthcare system.	5 (4.1)	6 (4.8)	23 (12.3)	4 (8.0)	38 (13.9)	14 (8.2)	477 (28.6)*	216 (12.1)	31 (15.0)	66 (14.0)	110 (13.8)
29. I worried about the infection situation.	6 (4.9)	8 (6.4)	49 (26.2)	4 (8.0)	59 (21.6)	39 (22.9)	606 (36.4)*	421 (23.5)	45 (21.8)	120 (25.5)	159 (19.9)
30. I worried about the spread of mutant strains such as the Delta variant.	4 (3.3)	1 (0.8)	12 (6.4)	3 (6.0)	49 (17.9)*	17 (10.0)	450 (27.0)*	220 (12.3)	21 (10.2)	64 (13.6)	105 (13.1)
31. I was beginning to think that vaccination is indispensable to contain the pandemic.	14 (11.5)	0 (0.0)	2 (1.1)	4 (8.0)	59 (21.6)*	12 (7.1)	397 (23.8)*	312 (17.4)	19 (9.2)	90 (19.1)	113 (14.1)

The asterisk refers to the reason answer choice that has the highest selection rate and is at least 5% higher than the second most commonly selected answer choice, and is interpreted as the overarching reason that characterizes each cluster. Group 1 refers to those who responded in the first survey that they did not intend to vaccinate and subsequently responded in the second survey that they were vaccinated or intending to be vaccinated. Group 2 refers to those who responded in the first survey that they were not sure whether or not to receive the vaccination and subsequently responded in the second survey that they were vaccinated or intending to be vaccinated.

Clusters on reasons for changes in vaccination intentions can be explained primarily by the following unique characteristics: perceived benefits of vaccination in general, awareness of the COVID-19 vaccination status of those close to oneself, work and personal relationship reasons, recognition of the social significance of COVID-19 vaccination for the spread of infection, and dispelled concerns about short-term adverse reactions and the safety of the COVID-19 vaccine.

For Group 2, we refer to the five clusters as follows: Cluster 1 (*n* = 1666, 33.8%) that considers the COVID-19 vaccine effective and socially significant with respect to controlling the spread of the infection, although short-term adverse reactions and safety concerns remain; Cluster 2 (*n* = 1791, 36.3%) with no clear overarching reasons; Cluster 3 (*n* = 206, 4.2%), for which concerns regarding future adverse reactions and the safety of COVID-19 vaccine and other vaccines in general have been dispelled and which also considered the COVID-19 vaccination status of people close to them; Cluster 4 (*n* = 471, 9.5%), for which there are still concerns about the effectiveness of the COVID-19 vaccine, but its benefits outweigh their doubts; and Cluster 5 (*n* = 799, 16.2%), for which concerns about short-term adverse reactions and the safety of the COVID-19 vaccine have been dispelled to some extent. Note that cluster numbers are not comparable between Group 1 and Group 2.

Clusters can be explained primarily by the following unique characteristics: perceived benefits of the COVID-19 vaccine, awareness of the COVID-19 vaccination status of those close to oneself, recognition of the social significance of COVID-19 vaccination for the spread of infection, and dispelled concerns about short-term adverse reactions and safety of the COVID-19 vaccine.

Supplementary Tables 1 and 2 show the distributions of the cluster-specific socio-demographic characteristics for Groups 1 and 2, respectively. For Group 1, age differed among clusters, with Cluster 5 having the highest. There were no meaningful differences in the distribution of other characteristics among clusters. For Group 2, there were differences among clusters with respect to age distribution, education level, occupation, household size, and marital status. A particularly clear trend was that there were relatively more people with lower education in Cluster 3 and more married and older people in Cluster 1.

Regarding the question about the Japanese government's COVID-19 health pass policy, 49.3% of the respondents who responded in the second survey that they have received or intended to receive the COVID-19 vaccine were in favour of the health pass, and 9.1% were against it. On the other hand, among those who responded in the second survey that they were unsure or did not intend to receive the COVID-19 vaccine, 9.6% were in favour of the health pass and 44.5% were against it. Among them, 7.5% responded that they would vaccinate if the health pass would change behaviour restrictions (Table 3).

**Table 3:** Responses to questions about the Japanese government's COVID-19 health pass policy.

	Total respondents in the second survey	Respondents (A) who indicated in the second survey that they have received or intended to receive the COVID-19 vaccine.	Respondents (B) who indicated in the second survey that they were unsure or did not intend to receive the COVID-19 vaccine
Do you support or oppose changing various activity restrictions depending on vaccination status (or whether or not one has proof of negative testing)?			
Support	8407 (43.8)	8152 (49.3)	255 (9.6)
Oppose	2681 (14.0)	1501 (9.1)	1180 (44.5)
Neither support nor oppose	8107 (42.2)	6892 (41.7)	1215 (45.8)
Total	19195	16545	2650
Do you receive a vaccine if various activity restrictions change depending on vaccination status (or whether or not one has proof of negative testing)?			
Yes	–	–	199 (7.5)
No	–	–	1305 (49.2)
Not sure	–	–	1146 (43.2)
Total	–	–	2650

## Discussion

There have been many studies on the determinants of intention to receive the COVID-19 vaccine. However, few studies have been conducted to investigate the reasons for changes in intention, such as the reversal of vaccine hesitancy. Our data showed that 46.9% and 80.9% of those who indicated in the first survey that they did not intend to or were unsure about receiving COVID-19 vaccination had received or showed willingness to receive the vaccine, respectively (72.6% when combined). Although it should be noted that follow-up periods vary from study to study, these vaccination rates are larger than those revealed in similar previous studies. In a study by Shaw et al. that tracked intention and actual COVID-19 vaccination status of refugees in the United States, 34.4% of those who did not intend to receive the vaccine and 55.8% of those who were unsure about receiving the vaccine got vaccinated or made a reservation to receive one within approximately six months.^13^ According to Olanipekun et al. that surveyed a cohort of patients who recovered from severe COVID-19 in the United States, 38.1% of those who responded that they would not accept a vaccine against COVID-19 and 15.4% of those who were undecided, changed their minds and received the vaccine within a year thereafter.^15^ Huang et al. also found that among people with multiple sclerosis in the United Kingdom, 24.7% and 28.6% of those who responded that they would definitely not and probably not get a COVID-19 vaccine, respectively, received the vaccine within a few months thereafter.^14^ On the other hand, a study by Evans et al. conducted on healthcare workers in the United States demonstrated that 59.7% of those who previously reported no intention of getting vaccinated against COVID-19 and 90.6% of those who had previously been unsure were vaccinated by follow-up six months later.^30^ These results are comparable to ours. The Understanding America Study (UAS), a repeated survey of a probabilistic Internet panel of adults in the United States, found that non-Hispanic Asian people were more likely to have increased likelihood of receiving a COVID-19 vaccine than non-Hispanic White people.^31^

We used a data clustering approach to characterize the overarching reasons for changes in vaccination intention from ‘unwilling to take a vaccine’ or ‘unsure’ to ‘accepting’ and found that there were six and five clusters, respectively. In Group 1, which initially indicated that they would not vaccinate against COVID-19, the clusters were characterized by the following: perceived benefits of vaccination in general, awareness of the COVID-19 vaccination status of those close to oneself, work and personal relationship reasons, recognition of the social significance of COVID-19 vaccination for the spread of infection, and dispelled concerns about short-term adverse reactions and the safety of the COVID-19 vaccine.

Our thorough literature review shows that few papers have evaluated the reasons for these changes in intention, but Olanipekun et al. found that knowing someone who received a COVID-19 vaccine and did not get sick afterwards resulted in a sense of security that the vaccine was safe, which led to a change in vaccine intention.^15^ This is consistent with the results of our study, which revealed a cluster of people who were vaccinated because someone close to them was vaccinated. The authors also state that wanting to protect others played an important role in changes in vaccine intention, which is also similar to the results of our study; social significance and concerns about the spread of infection were identified as overarching reasons characterizing a cluster.^15^ In addition, the authors recommend that employers encourage their employees to vaccinate against COVID-19. Our study detected a cluster of respondents who were vaccinated in order to facilitate their work, which supports this recommendation.^15^

Similar cluster characteristics were also detected in Group 2, comprised of those who initially responded that they were unsure about vaccination against COVID-19. However, no cluster was identified as having been vaccinated for the purpose of facilitating work. For those who are unsure about vaccination, workplace recommendations may not always be effective. The Group 1 and Group 2 clusters have similarities in their distribution of respondents: the cluster with no clear overarching reason had the largest number of respondents in both Cluster 3 and Cluster 2, respectively. Similarly, there were more respondents in the clusters where recognition of the social significance of the COVID-19 vaccine and concerns about the spread of infection were overarching reasons, and respondents were older than in the other clusters (Cluster 5 and Cluster 1, respectively).

It is interesting to note that previous studies from the United States and Austria found that recommendations from healthcare providers played an important role in changing vaccination intentions with regard to COVID-19 and influenza vaccines.^15^^,^^32^ However, no clusters with that overarching reason were detected in either Group 1 or Group 2 in the present study.

Governments and politicians in different countries will make and have made different choices about policies regarding the COVID-19 vaccine, including health passes. They consider the sustainability of their respective healthcare systems and the socio-economic impact of the pandemic. A study in the United Kingdom by Figueiredo et al. showed that the health pass was supported by those who were already positive about vaccination, but increased hesitancy to vaccination among those who had less confidence in the vaccine.^33^ In our study, 9.1% of the respondents who reported in the second survey that they have received or showed willingness to receive a COVID-19 vaccine disagreed with the health pass. Meanwhile, 44.5% of those who responded in the second survey that they were unsure or did not intend to receive the COVID-19 vaccine disagreed; and only 7.5% of those respondents answered that they would be vaccinated if health passes were implemented and would change behaviour restrictions. It is important to recognize that while a concerted effort is needed to encourage vaccine uptake and reach those who are hesitant, the introduction of mandatory COVID-19 certification or vaccine passports risks exacerbating inequalities among communities with low vaccination coverage.^34^^,^^35^

### Limitations

As already reported elsewhere,^10^^,^^36^ the survey data used in this study have several limitations. First, since policies and progress on COVID-19 vaccination vary from country to country, the findings of this study in the Japanese context are not necessarily applicable to other countries or regions. Second, self-selection bias affects the representativeness of survey participants, but the survey has a mechanism that provides an incentive to respond even to those who are not interested in the survey by awarding points (by the survey company) that can be used to purchase products from the survey company's partners. In addition, because survey participants were recruited using a quota sampling method based on age, gender, and prefecture population ratios from the 2015 National Census, the first survey was fairly representative of Japan's population, except that the education level of the respondents was relatively higher than those identified in the Census. The second survey used the same methodology with the same issues arising with respect to education. Since higher levels of education have been suggested to be associated with being more likely to be vaccinated against COVID-19,^19^^,^^20^ it is possible that the reversal rate of COVID-19 vaccination hesitancy identified in the present study was overestimated. However, the impact of this bias on our cluster analysis is unknown because of the lack of evidence regarding the relationship between education level and reasons for the reversal. As of February 24, 2022, when the second survey was completed, vaccination coverage for the entire Japanese population was (75.2%), and for those aged 65 years and older, the corresponding rate was 92.7%.^5^ Similarly, our data demonstrated that vaccination coverage among the second survey respondents (aged 20 years and older) was 84.9% and that for the respondents aged 65 years and older was 93.1%, suggesting that the second survey respondents are highly nationally representative in terms of vaccination coverage. Third, the 31 reasons for change in vaccination intention utilized in this study were our own, based on prior research findings and in consultation between authors who are in a position to support COVID-19 vaccine policy development and clinical policy. Although we considered a variety of possible themes, the diversity of reasons in a multiple-response format will necessarily be limited compared to those received using an open-ended response format. However, the study setting of the second, follow-up survey taking place one year after vaccination was initiated in Japan may contribute significantly to recall bias, so we believe that a closed-ended format is most appropriate. Fourth, although we defined ‘reversal of vaccine hesitancy’ based in part on vaccination behaviour, the latest research has proposed that the concept of hesitancy is not necessarily reflected in vaccination behaviour, which is influenced more by physical accessibility to vaccination or convenience. However, our data were obtained from a survey conducted well after the start of COVID-19 vaccination, and thus, we believe that convenience has not significantly affected respondents’ vaccination behaviour, and the concept of hesitancy proposed by Bedford et al.^27^ and ours are nearly synonymous. Finally, two heuristic choices were made in this study. The first was the determination of a reachable distance threshold that separates the clusters. Following the guidelines for use of OPTICS and others,^25^^,^^26^ we selected the most reasonable threshold at which clusters can be visually discriminated from each other. Second, we set a threshold of 5% for the difference in response rates between clusters for each reason option and judged that exceeding this threshold was indicative of an important characteristic of the cluster. If the threshold is smaller or larger than 5%, it is not possible to clearly distinguish the difference in characteristics from the other clusters. For example, there are not many reason options with response rates above 5%.

## Conclusions

This study provides important evidence as to why people in Japan who did not intend to be vaccinated against COVID-19 or were unsure changed their minds and were accepting of the vaccine by follow-up one year later. Our data demonstrated that the intention to vaccinate among the Japanese is more variable than previous studies in other countries have shown. The overarching reasons for reversal of vaccine hesitancy were characterized by the perceived benefits of vaccination, including the COVID-19 vaccine, awareness of the COVID-19 vaccination status of those close to them, recognition of the social significance of COVID-19 vaccination for the spread of infection, and dispelled concerns about short-term adverse reactions and the safety of the COVID-19 vaccine. Only for those who did not intend to vaccinate, work and personal relationship reasons were also found to be a unique overarching reason for vaccination changes of heart. Successful global vaccination promotion efforts will require increased efforts to determine how to get people to accept vaccines. The results of this study will serve as one piece of evidence to improve the effectiveness of public health measures.

## Contributors

Conception/design of the work: S.N., A.E., D.Y., M.M., Y.T., T.Y., T.K., K.M.S., S.G., H.K., S.K., and H.M.; acquisition of data: S.N., K.M.S., H.K., S.K., K.S., and H.M.; analysis of data: S.N., A.E., D.Y., Y.T., T.K.; interpretation of findings: all authors; drafting of the work: S.N.; substantially revised the work: S.N., A.E., D.Y., M.M., T.Y., A.T., H.S., S.G., and Y.Y.

## Data sharing statement

The datasets generated during and/or analysed during the current study are not publicly available due to ethical considerations but are available from the corresponding author on reasonable request.

## Ethics statement

Ethical approval was granted by the Ethics Committee of Keio University School of Medicine under authorization number 20200340. Respondents had to provide their consent before they proceeded to the questionnaire response page.

## Declaration of interests

Arata Takahashi and Hiroaki Miyata are affiliated with the Department of Healthcare Quality Assessment at The University of Tokyo. The department is a social collaboration department supported by grants from the National Clinical Database, Johnson & Johnson K.K., and Nipro Co. The remaining authors declare no conflicts of interest for this article.

## Appendix Supplementary materials

Image, application 1
