# Supplementary material for: Characterising reasons for reversals of COVID-19 vaccination hesitancy among Japanese people: One-year follow-up survey
Source: Lancet Reg Health West Pac. 2022 Jul 21;27:100541. doi: 10.1016/j.lanwpc.2022.100541 (PMC9302916; doi:10.1016/j.lanwpc.2022.100541)
Supplement: Supplementary file 1 [file mmc1.pdf]

Supplementary text: Dimension reduction by Uniform Manifold Approximation and Projection (UMAP)

McInnes *et al*. (2018) developed UMAP (Uniform Manifold Approximation and Projection),[1] which falls under the class of (unsupervised) manifold learning techniques for dimension reduction based on Riemannian geometry and algebraic topology. Compared with famous and widely used dimension reduction techniques such as t-SNE (t-Distributed Stochastic Neighbor Embedding) and PCA (Principal Component Analysis), UMAP offers some advantages with respect to increased speed and better preservation of the data's global structure in lower dimensions.

UMAP consists of two steps: construction of a (weighted) graph in high dimensions that represents the dataset topology and then optimization of the associated cost function to construct the most similar graph in lower dimensions. The graph node represents a datapoint and the edge represents how close a given set of points is using the distance measure on the manifold. In lower dimensions, UMAP finds *k* nearest neighbours on an *n*-dimensional manifold based on the topology of the data. Detailed algorithms can be found in McInnes *et al*. (2018),[1] and the *uwot* package in R provides several functions for the implementation of UMAP. In this study, we mapped the 31 binary variable into two-dimensional space (manifold) followed by visual inspection. Since all variables are binary, hamming distance was used as the distance metric on the manifold. Also, we use *k*=15 for identifying neighborhoods on the graph. Other settings were set to the *uwo*'package's default values.

Reference

1. McInnes L, Healy J. UMAP: Uniform Manifold Approximation and Projection for dimension reduction. *ArXiv* 2018; **abs/1802.03426**.

Supplementary Figure 1: Reachability plots for Groups 1 and 2, generated by OPTICS algorithm in clustering reduced-dimensional data

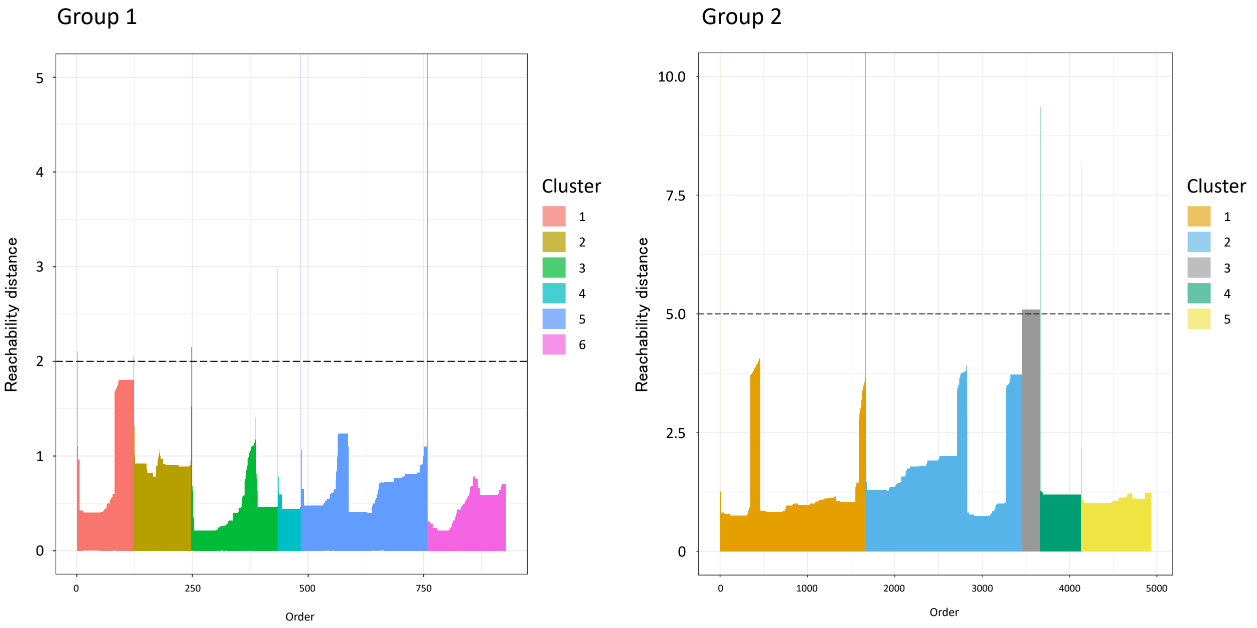

Group 1 refers to those who responded in the first survey that they did not intend to vaccinate and subsequently responded in the second survey that they were vaccinated or intending to be vaccinated. Group 2 refers to those who responded in the first survey that they were not sure whether or not to receive the vaccination and subsequently responded in the second survey that they were vaccinated or intending to be vaccinated. Note that cluster numbers are not comparable between Group 1 and Group 2.

Supplementary Table 1: Socio-demographic characteristics of Group 1 by clusters: number (%)

	Cluster 1	Cluster 2	Cluster 3	Cluster 4	Cluster 5	Cluster 6
Number of respondents	122 (13.2)	125 (13.5)	187 (20.2)	50 (5.4)	273 (29.4)	170 (18.3)
Age (standard deviation)	48.1 (14.9)	44.1 (15.0)	44.5 (14.5)	46.7 (11.8)	50.6 (15.3)	46.7 (14.7)
Prefecture (SA)						
Hokkaido	5 (4.1)	10 (8.0)	9 (4.8)	2 (4.0)	16 (5.9)	10 (5.9)
Aomori	1 (0.8)	0 (0.0)	2 (1.1)	2 (4.0)	2 (0.7)	3 (1.8)
Iwate	1 (0.8)	1 (0.8)	2 (1.1)	1 (2.0)	3 (1.1)	1 (0.6)
Miyagi	1 (0.8)	2 (1.6)	3 (1.6)	0 (0.0)	5 (1.8)	3 (1.8)
Akita	1 (0.8)	1 (0.8)	2 (1.1)	0 (0.0)	0 (0.0)	0 (0.0)
Yamagata	0 (0.0)	0 (0.0)	2 (1.1)	0 (0.0)	3 (1.1)	0 (0.0)
Fukushima	1 (0.8)	2 (1.6)	1 (0.5)	0 (0.0)	3 (1.1)	2 (1.2)
Ibaraki	0 (0.0)	2 (1.6)	3 (1.6)	2 (4.0)	6 (2.2)	3 (1.8)
Tochigi	1 (0.8)	1 (0.8)	1 (0.5)	0 (0.0)	1 (0.4)	2 (1.2)
Gunma	1 (0.8)	0 (0.0)	1 (0.5)	1 (2.0)	1 (0.4)	0 (0.0)
Saitama	7 (5.7)	9 (7.2)	20 (10.7)	2 (4.0)	16 (5.9)	9 (5.3)
Chiba	7 (5.7)	3 (2.4)	6 (3.2)	4 (8.0)	14 (5.1)	5 (2.9)
Tokyo	17 (13.9)	21 (16.8)	34 (18.2)	7 (14.0)	36 (13.2)	23 (13.5)
Kanagawa	4 (3.3)	8 (6.4)	6 (3.2)	5 (10.0)	24 (8.8)	16 (9.4)
Niigata	6 (4.9)	0 (0.0)	0 (0.0)	0 (0.0)	5 (1.8)	2 (1.2)
Toyama	0 (0.0)	3 (2.4)	2 (1.1)	1 (2.0)	1 (0.4)	1 (0.6)
Ishikawa	0 (0.0)	0 (0.0)	0 (0.0)	1 (2.0)	0 (0.0)	1 (0.6)
Fukui	1 (0.8)	0 (0.0)	1 (0.5)	0 (0.0)	1 (0.4)	0 (0.0)
Yamanashi	0 (0.0)	3 (2.4)	1 (0.5)	0 (0.0)	1 (0.4)	1 (0.6)
Nagano	4 (3.3)	2 (1.6)	4 (2.1)	0 (0.0)	5 (1.8)	3 (1.8)
Gifu	1 (0.8)	4 (3.2)	4 (2.1)	1 (2.0)	4 (1.5)	2 (1.2)
Shizuoka	5 (4.1)	1 (0.8)	2 (1.1)	1 (2.0)	3 (1.1)	7 (4.1)
Aichi	10 (8.2)	9 (7.2)	11 (5.9)	5 (10.0)	18 (6.6)	9 (5.3)
Mie	0 (0.0)	1 (0.8)	3 (1.6)	1 (2.0)	5 (1.8)	2 (1.2)
Shiga	1 (0.8)	0 (0.0)	2 (1.1)	1 (2.0)	2 (0.7)	0 (0.0)
Kyoto	1 (0.8)	4 (3.2)	6 (3.2)	0 (0.0)	7 (2.6)	4 (2.4)
Osaka	9 (7.4)	5 (4.0)	10 (5.3)	5 (10.0)	20 (7.3)	10 (5.9)
Hyogo	11 (9.0)	3 (2.4)	10 (5.3)	2 (4.0)	11 (4.0)	3 (1.8)
Nara	4 (3.3)	2 (1.6)	1 (0.5)	2 (4.0)	5 (1.8)	4 (2.4)
Wakayama	0 (0.0)	1 (0.8)	3 (1.6)	0 (0.0)	2 (0.7)	1 (0.6)
Tottori	0 (0.0)	1 (0.8)	1 (0.5)	0 (0.0)	2 (0.7)	0 (0.0)
Shimane	0 (0.0)	0 (0.0)	0 (0.0)	0 (0.0)	0 (0.0)	1 (0.6)
Okayama	1 (0.8)	2 (1.6)	3 (1.6)	0 (0.0)	7 (2.6)	4 (2.4)
Hiroshima	4 (3.3)	3 (2.4)	3 (1.6)	0 (0.0)	6 (2.2)	6 (3.5)
Yamaguchi	1 (0.8)	0 (0.0)	0 (0.0)	0 (0.0)	0 (0.0)	1 (0.6)
Tokushima	2 (1.6)	0 (0.0)	2 (1.1)	0 (0.0)	2 (0.7)	1 (0.6)
Kagawa	0 (0.0)	2 (1.6)	2 (1.1)	0 (0.0)	3 (1.1)	2 (1.2)
Ehime	2 (1.6)	0 (0.0)	1 (0.5)	0 (0.0)	3 (1.1)	4 (2.4)
Kochi	1 (0.8)	1 (0.8)	0 (0.0)	0 (0.0)	1 (0.4)	0 (0.0)
Fukuoka	6 (4.9)	9 (7.2)	13 (7.0)	4 (8.0)	8 (2.9)	13 (7.6)
Saga	0 (0.0)	1 (0.8)	1 (0.5)	0 (0.0)	0 (0.0)	0 (0.0)
Nagasaki	0 (0.0)	1 (0.8)	1 (0.5)	0 (0.0)	4 (1.5)	3 (1.8)
Kumamoto	1 (0.8)	3 (2.4)	2 (1.1)	0 (0.0)	3 (1.1)	3 (1.8)
Oita	0 (0.0)	1 (0.8)	2 (1.1)	0 (0.0)	3 (1.1)	1 (0.6)
Miyazaki	1 (0.8)	0 (0.0)	0 (0.0)	0 (0.0)	3 (1.1)	1 (0.6)
Kagoshima	2 (1.6)	2 (1.6)	1 (0.5)	0 (0.0)	7 (2.6)	2 (1.2)
Okinawa	1 (0.8)	1 (0.8)	3 (1.6)	0 (0.0)	1 (0.4)	1 (0.6)
Gender (SA)						
Women	64 (52.5)	68 (54.4)	92 (49.2)	21 (42.0)	146 (53.5)	85 (50.0)
Men	58 (47.5)	57 (45.6)	94 (50.3)	29 (58.0)	127 (46.5)	85 (50.0)
Other	0 (0.0)	0 (0.0)	1 (0.5)	0 (0.0)	0 (0.0)	0 (0.0)
Highest educational level (SA)						
Middle school	4 (3.3)	2 (1.6)	5 (2.7)	0 (0.0)	7 (2.6)	1 (0.6)
High school	35 (28.7)	35 (28.0)	52 (27.8)	20 (40.0)	88 (32.2)	60 (35.3)
Junior college	26 (21.3)	24 (19.2)	35 (18.7)	4 (8.0)	52 (19.0)	28 (16.5)
University	52 (42.6)	54 (43.2)	85 (45.5)	22 (44.0)	112 (41.0)	72 (42.4)
Graduate school (master's course)	4 (3.3)	9 (7.2)	8 (4.3)	3 (6.0)	12 (4.4)	7 (4.1)

Graduate school (doctoral course)	1 (0.8)	1 (0.8)	2 (1.1)	1 (2.0)	2 (0.7)	2 (1.2)
Occupation type (SA)						
Healthcare workers	3 (2.5)	7 (5.6)	9 (4.8)	6 (12.0)	16 (9.4)	17 (6.2)
Social and education workers	14 (11.5)	13 (10.4)	29 (15.5)	2 (4.0)	19 (11.2)	24 (8.8)
Other essential workers	51 (41.8)	59 (47.2)	74 (39.6)	26 (52.0)	72 (42.4)	91 (33.3)
Non-essential workers	39 (32.0)	33 (26.4)	55 (29.4)	13 (26.0)	40 (23.5)	94 (34.4)
Other	15 (12.3)	13 (10.4)	20 (10.7)	3 (6.0)	23 (13.5)	47 (17.2)
Annual household income in 2020 – million JPY (SA)						
–1)	10 (8.2)	10 (8.0)	15 (8.0)	2 (4.0)	19 (7.0)	13 (7.6)
[1–2)	16 (13.1)	13 (10.4)	21 (11.2)	4 (8.0)	28 (10.3)	15 (8.8)
[2–3)	15 (12.3)	19 (15.2)	16 (8.6)	4 (8.0)	46 (16.8)	24 (14.1)
[3–4)	18 (14.8)	15 (12.0)	25 (13.4)	11 (22.0)	36 (13.2)	21 (12.4)
[4–5)	15 (12.3)	18 (14.4)	22 (11.8)	5 (10.0)	28 (10.3)	21 (12.4)
[5–6)	13 (10.7)	9 (7.2)	19 (10.2)	4 (8.0)	24 (8.8)	18 (10.6)
[6–7)	4 (3.3)	11 (8.8)	20 (10.7)	6 (12.0)	20 (7.3)	13 (7.6)
[7–8)	10 (8.2)	5 (4.0)	16 (8.6)	7 (14.0)	20 (7.3)	16 (9.4)
[8–9)	4 (3.3)	2 (1.6)	8 (4.3)	1 (2.0)	13 (4.8)	13 (7.6)
[9–10)	2 (1.6)	12 (9.6)	7 (3.7)	2 (4.0)	6 (2.2)	6 (3.5)
[10– or	15 (12.3)	11 (8.8)	18 (9.6)	4 (8.0)	33 (12.1)	10 (5.9)
Household size including respondent (SA)						
1	27 (22.1)	33 (26.4)	36 (19.3)	13 (26.0)	51 (18.7)	44 (25.9)
2	39 (32.0)	38 (30.4)	62 (33.2)	17 (34.0)	101 (37.0)	55 (32.4)
3	27 (22.1)	30 (24.0)	43 (23.0)	10 (20.0)	67 (24.5)	41 (24.1)
4	22 (18.0)	17 (13.6)	30 (16.0)	8 (16.0)	36 (13.2)	21 (12.4)
5	3 (2.5)	4 (3.2)	9 (4.8)	2 (4.0)	12 (4.4)	8 (4.7)
More than 6	4 (3.3)	3 (2.4)	7 (3.7)	0 (0.0)	6 (2.2)	1 (0.6)
Marital size (SA)						
Married (including de facto marriage)	63 (51.6)	56 (44.8)	97 (51.9)	26 (52.0)	159 (58.2)	82 (48.2)
Not married (without partner)	32 (26.2)	41 (32.8)	62 (33.2)	17 (34.0)	70 (25.6)	58 (34.1)
Not married (with a partner)	7 (5.7)	14 (11.2)	14 (7.5)	4 (8.0)	18 (6.6)	10 (5.9)
Widowed	5 (4.1)	5 (4.0)	3 (1.6)	0 (0.0)	5 (1.8)	8 (4.7)
Divorced	15 (12.3)	9 (7.2)	11 (5.9)	3 (6.0)	21 (7.7)	12 (7.1)
No underlying diseases (SA)	107 (87.7)	114 (91.2)	171 (91.4)	48 (96.0)	252 (92.3)	160 (94.1)

Group 1 refers to those who responded in the first survey that they did not intend to vaccinate and subsequently responded in the second survey that they were vaccinated or intending to be vaccinated. SA refers to single-answer questions. Underling diseases include diabetes, heart failure, respiratory disease, COPD, etc., being on dialysis, or using immunosuppressive or anticancer therapies. Data are as of the first survey, except for prefecture of residence.

Supplementary Table 2: Socio-demographic characteristics of Group 2 by clusters: number (%)

	Cluster 1	Cluster 2	Cluster 3	Cluster 4	Cluster 5
Number of respondents	1666 (33.8)	1791 (36.3)	206 (4.2)	471 (9.5)	799 (16.2)
Age (standard deviation)	55.3 (14.4)	49.3 (15.0)	54.1 (15.4)	54.4 (14.7)	54.1 (15.2)
Prefecture (SA)					
Hokkaido	98 (5.9)	92 (5.1)	14 (6.8)	25 (5.3)	41 (5.1)
Aomori	16 (1.0)	17 (0.9)	1 (0.5)	3 (0.6)	6 (0.8)
Iwate	15 (0.9)	13 (0.7)	4 (1.9)	2 (0.4)	8 (1.0)
Miyagi	35 (2.1)	36 (2.0)	2 (1.0)	8 (1.7)	10 (1.3)
Akita	13 (0.8)	10 (0.6)	1 (0.5)	4 (0.8)	5 (0.6)
Yamagata	8 (0.5)	9 (0.5)	3 (1.5)	1 (0.2)	7 (0.9)
Fukushima	15 (0.9)	20 (1.1)	3 (1.5)	4 (0.8)	6 (0.8)
Ibaraki	28 (1.7)	24 (1.3)	5 (2.4)	2 (0.4)	18 (2.3)
Tochigi	18 (1.1)	16 (0.9)	0 (0.0)	4 (0.8)	6 (0.8)
Gunma	12 (0.7)	18 (1.0)	1 (0.5)	3 (0.6)	8 (1.0)
Saitama	100 (6.0)	101 (5.6)	18 (8.7)	26 (5.5)	55 (6.9)
Chiba	71 (4.3)	86 (4.8)	9 (4.4)	25 (5.3)	42 (5.3)
Tokyo	187 (11.2)	199 (11.1)	23 (11.2)	52 (11.0)	84 (10.5)
Kanagawa	160 (9.6)	163 (9.1)	8 (3.9)	35 (7.4)	82 (10.3)
Niigata	29 (1.7)	39 (2.2)	5 (2.4)	7 (1.5)	16 (2.0)
Toyama	17 (1.0)	14 (0.8)	2 (1.0)	3 (0.6)	5 (0.6)
Ishikawa	11 (0.7)	18 (1.0)	3 (1.5)	2 (0.4)	8 (1.0)
Fukui	10 (0.6)	9 (0.5)	2 (1.0)	5 (1.1)	3 (0.4)
Yamanashi	14 (0.8)	7 (0.4)	0 (0.0)	2 (0.4)	1 (0.1)
Nagano	34 (2.0)	39 (2.2)	1 (0.5)	8 (1.7)	17 (2.1)
Gifu	30 (1.8)	14 (0.8)	2 (1.0)	5 (1.1)	15 (1.9)
Shizuoka	41 (2.5)	43 (2.4)	6 (2.9)	20 (4.2)	19 (2.4)
Aichi	96 (5.8)	112 (6.3)	11 (5.3)	34 (7.2)	43 (5.4)
Mie	20 (1.2)	24 (1.3)	2 (1.0)	5 (1.1)	14 (1.8)
Shiga	10 (0.6)	9 (0.5)	0 (0.0)	4 (0.8)	11 (1.4)
Kyoto	34 (2.0)	36 (2.0)	7 (3.4)	6 (1.3)	13 (1.6)
Osaka	133 (8.0)	155 (8.7)	13 (6.3)	34 (7.2)	61 (7.6)
Hyogo	71 (4.3)	80 (4.5)	14 (6.8)	25 (5.3)	41 (5.1)
Nara	14 (0.8)	21 (1.2)	1 (0.5)	4 (0.8)	8 (1.0)
Wakayama	3 (0.2)	10 (0.6)	2 (1.0)	2 (0.4)	5 (0.6)
Tottori	8 (0.5)	8 (0.4)	1 (0.5)	3 (0.6)	1 (0.1)
Shimane	4 (0.2)	4 (0.2)	2 (1.0)	2 (0.4)	1 (0.1)
Okayama	31 (1.9)	26 (1.5)	3 (1.5)	9 (1.9)	14 (1.8)
Hiroshima	45 (2.7)	42 (2.3)	4 (1.9)	13 (2.8)	17 (2.1)
Yamaguchi	15 (0.9)	19 (1.1)	0 (0.0)	6 (1.3)	11 (1.4)
Tokushima	14 (0.8)	13 (0.7)	0 (0.0)	2 (0.4)	2 (0.3)
Kagawa	15 (0.9)	22 (1.2)	2 (1.0)	4 (0.8)	4 (0.5)
Ehime	18 (1.1)	18 (1.0)	3 (1.5)	6 (1.3)	9 (1.1)
Kochi	5 (0.3)	3 (0.2)	2 (1.0)	3 (0.6)	4 (0.5)
Fukuoka	71 (4.3)	97 (5.4)	13 (6.3)	33 (7.0)	42 (5.3)
Saga	11 (0.7)	11 (0.6)	1 (0.5)	5 (1.1)	2 (0.3)
Nagasaki	14 (0.8)	20 (1.1)	2 (1.0)	7 (1.5)	8 (1.0)
Kumamoto	18 (1.1)	13 (0.7)	5 (2.4)	6 (1.3)	6 (0.8)
Oita	10 (0.6)	11 (0.6)	4 (1.9)	4 (0.8)	1 (0.1)
Miyazaki	10 (0.6)	16 (0.9)	0 (0.0)	3 (0.6)	5 (0.6)
Kagoshima	14 (0.8)	14 (0.8)	1 (0.5)	3 (0.6)	5 (0.6)
Okinawa	20 (1.2)	20 (1.1)	0 (0.0)	2 (0.4)	9 (1.1)
Gender (SA)					
Women	962 (57.7)	1009 (56.3)	125 (60.7)	252 (53.5)	451 (56.4)
Men	703 (42.2)	780 (43.6)	81 (39.3)	219 (46.5)	348 (43.6)
Other	1 (0.1)	2 (0.1)	0 (0.0)	0 (0.0)	0 (0.0)
Highest educational level (SA)					
Middle school	35 (2.1)	57 (3.2)	5 (2.4)	19 (4.0)	25 (3.1)
High school	588 (35.3)	623 (34.8)	82 (39.8)	176 (37.4)	297 (37.2)
Junior college	348 (20.9)	386 (21.6)	59 (28.6)	91 (19.3)	169 (21.2)
University	636 (38.2)	657 (36.7)	56 (27.2)	166 (35.2)	290 (36.3)
Graduate school (master's course)	43 (2.6)	61 (3.4)	4 (1.9)	16 (3.4)	12 (1.5)

Graduate school (doctoral course)	16 (1.0)	7 (0.4)	0 (0.0)	3 (0.6)	6 (0.8)
Occupation type (SA)					
Healthcare workers	55 (3.3)	89 (5.0)	7 (3.4)	27 (5.7)	37 (4.6)
Social and education workers	139 (8.3)	201 (11.2)	20 (9.7)	43 (9.1)	54 (6.8)
Other essential workers	582 (34.9)	692 (38.6)	76 (36.9)	156 (33.1)	293 (36.7)
Non-essential workers	576 (34.6)	541 (30.2)	66 (32.0)	143 (30.4)	267 (33.4)
Other	314 (18.8)	268 (15.0)	37 (18.0)	102 (21.7)	148 (18.5)
Annual household income in 2020 – million JPY (SA)					
–1)	98 (5.9)	146 (8.2)	14 (6.8)	37 (7.9)	56 (7.0)
[1–2)	143 (8.6)	172 (9.6)	22 (10.7)	42 (8.9)	81 (10.1)
[2–3)	261 (15.7)	235 (13.1)	27 (13.1)	81 (17.2)	112 (14.0)
[3–4)	276 (16.6)	269 (15.0)	26 (12.6)	78 (16.6)	143 (17.9)
[4–5)	201 (12.1)	228 (12.7)	31 (15.0)	52 (11.0)	94 (11.8)
[5–6)	166 (10.0)	190 (10.6)	30 (14.6)	42 (8.9)	93 (11.6)
[6–7)	136 (8.2)	145 (8.1)	9 (4.4)	37 (7.9)	57 (7.1)
[7–8)	116 (7.0)	128 (7.1)	9 (4.4)	22 (4.7)	36 (4.5)
[8–9)	63 (3.8)	67 (3.7)	12 (5.8)	14 (3.0)	39 (4.9)
[9–10)	65 (3.9)	68 (3.8)	8 (3.9)	21 (4.5)	31 (3.9)
[10– or	141 (8.5)	143 (8.0)	18 (8.7)	45 (9.6)	57 (7.1)
Household size including respondent (SA)					
1	287 (17.2)	353 (19.7)	30 (14.6)	94 (20.0)	129 (16.1)
2	650 (39.0)	553 (30.9)	84 (40.8)	182 (38.6)	330 (41.3)
3	408 (24.5)	463 (25.9)	50 (24.3)	97 (20.6)	173 (21.7)
4	238 (14.3)	302 (16.9)	28 (13.6)	75 (15.9)	129 (16.1)
5	52 (3.1)	75 (4.2)	6 (2.9)	17 (3.6)	26 (3.3)
More than 6	31 (1.9)	45 (2.5)	8 (3.9)	6 (1.3)	12 (1.5)
Marital size (SA)					
Married (including de facto marriage)	1057 (63.4)	954 (53.3)	111 (53.9)	287 (60.9)	493 (61.7)
Not married (without partner)	367 (22.0)	543 (30.3)	40 (19.4)	117 (24.8)	181 (22.7)
Not married (with a partner)	65 (3.9)	118 (6.6)	15 (7.3)	13 (2.8)	34 (4.3)
Widowed	82 (4.9)	62 (3.5)	11 (5.3)	17 (3.6)	33 (4.1)
Divorced	95 (5.7)	114 (6.4)	29 (14.1)	37 (7.9)	58 (7.3)
No underlying diseases (SA)	1482 (89.0)	1621 (90.5)	186 (90.3)	406 (86.2)	709 (88.7)

Group 2 refers to those who responded in the first survey that they were not sure whether or not to receive the vaccination and subsequently responded in the second survey that they were vaccinated or intending to be vaccinated. SA refers to single-answer questions. Underling diseases include diabetes, heart failure, respiratory disease, COPD, etc., being on dialysis, or using immunosuppressive or anticancer therapies. Data are as of the first survey, except for prefecture of residence.

Original Japanese questionnaire

Q1 インターネットを用いた調査においては、うそをついたり、質問を読まないで、いい加減な回答をしたりする方がいることが問題となっています。あなたは質問をきちんと読んで、真面目に答えていただけますか？真面目に答えていただけるのであれば、以下のボックスをチェックしていただき、次のページへお進みください。（SA）

1 私は以下の質問をきちんと読んで、真面目に回答します
2 回答を辞退する

Q2 あなたのお住まいの都道府県を教えてください。（SA）

1 北海道
2 青森県
3 岩手県
4 宮城県
5 秋田県
6 山形県
7 福島県
8 茨城県
9 栃木県
10 群馬県
11 埼玉県
12 千葉県
13 東京都
14 神奈川県
15 新潟県
16 富山県
17 石川県
18 福井県
19 山梨県
20 長野県
21 岐阜県
22 静岡県
23 愛知県
24 三重県
25 滋賀県
26 京都府
27 大阪府
28 兵庫県
29 奈良県

30 和歌山県
31 鳥取県
32 島根県
33 岡山県
34 広島県
35 山口県
36 徳島県
37 香川県
38 愛媛県
39 高知県
40 福岡県
41 佐賀県
42 長崎県
43 熊本県
44 大分県
45 宮崎県
46 鹿児島県
47 沖縄県

Q3 現在の健康状態はいかがですか？（SA）

1 よい
2 まあよい
3 ふつう
4 あまりよくない
5 よくない

Q4 医療機関受診時、自身の健康状態を予診票に記入する際にどの程度自信をもって記入できますか？（SA）

1 全くできない
2 できない
3 どちらともいえない
4 できる
5 十分できる

Q5 新型コロナウイルスの検査を受けたことがありますか？（MA）

1 いいえ
2 PCR検査を受けたことがある
3 抗原検査を受けたことがある

4　抗体検査を受けたことがある
5　いずれかを受けたがどれかは覚えていない

Q6　新型コロナウイルスについて、どの程度不安がありますか？（SA）

1　全然不安はない
2　漠然とした不安がある
3　明確に不安を感じている
4　不安に加え恐怖を感じている

Q7　この１年間に、新型コロナウイルスの流行により、あなたの生活への支障はどの程度ありましたか？（SA）

1　全くなかった
2　あまりなかった
3　ある程度あった
4　非常にあった

Q8　新型コロナウイルス感染予防のために、していることをお選びください。（MA）

1　換気が悪い場所には行かないようにしている
2　人がたくさん集まっている場所には行かないようにしている
3　他の人と、近い距離での会話や発声をしないようにしている
4　手洗い・うがいやアルコールによる手や指の消毒をしている
5　せきやくしゃみをする時は、マスク・ハンカチ等を口にあてる
6　仕事はテレワークにしている
7　社会的距離を意識して行動している
8　外出時はマスクを着用している
9　大人数での会食や飲み会を避けている
10　同居する家族以外との食事は極力避けている
11　こまめな換気を行っている
12　不要不急の外出を避ける
13　その他
14　特にやっていることはない

Q9　今後半年以内に新型コロナウイルスに感染するか否かについて、あなたの推測を教えてください。（SA）

1　新型コロナウイルスには感染しないと思う
2　新型コロナウイルスに感染すると思うが重症化はしないと思う
3　新型コロナウイルスに感染し、重症化すると思う
4　すでに新型コロナウイルスに感染したことがある

Q10　身近な方に新型コロナウイルスに感染した経験がある方はいらっしゃいますか？またはご存知ですか？（MA）

1　家族（軽症）
2　家族（中等症）
3　家族（重症）
4　家族（症状はわからない）
5　友人や職場の同僚（軽症）
6　友人や職場の同僚（中等症）
7　友人や職場の同僚（重症）
8　友人や職場の同僚（症状はわからない）
9　いない／わからない

Q11　新型コロナウイルスに関して、どのような情報源から情報を得ていますか？（MA）

1　医師
2　看護師
3　薬剤師
4　獣医
5　歯科医
6　健康フェア・イベント
7　新聞
8　雑誌
9　本
10　科学文献（論文）
11　テレビ
12　ラジオ
13　ネットのニュースサイト
14　検索エンジン（Google、Yahooなど）
15　LINE／ライン
16　Facebook／フェイスブック
17　Twitter／ツイッター
18　Instagram／インスタグラム
19　YouTube／ユーチューブ
20　TikTok／ティックトック
21　医療系情報サイト（Medical Note、MEDLEY 、アピタルなど）
22　ブログまたは有名人、著名人のウェブページ
23　都道府県や市町村などの自治体
24　政府
25　新型コロナウイルス感染症対策専門家会議（現分科会）

26 友達

27 家族

28 科学者や研究者

29 製薬企業

30 他の会社または企業

Q12 新型コロナウイルスに関する情報源として、以下の情報源からの情報をどの程度信頼していますか？それぞれ4段階でお選びください。（SA）

1 医師

2 看護師

3 薬剤師

4 獣医

5 歯科医

6 健康フェア・イベント

7 新聞

8 雑誌

9 本

10 科学文献（論文）

11 テレビ

12 ラジオ

13 ネットのニュースサイト

14 検索エンジン（Google、Yahooなど）

15 LINE／ライン

16 Facebook／フェイスブック

17 Twitter／ツイッター

18 Instagram／インスタグラム

19 YouTube／ユーチューブ

20 TikTok／ティックトック

21 医療系情報サイト（Medical Note、MEDLEY 、アピタルなど）

22 ブログまたは有名人、著名人のウェブページ

23 都道府県や市町村などの自治体

24 政府

25 新型コロナウイルス感染症対策専門家会議（現分科会）

26 友達

27 家族

28 科学者や研究者

29 製薬企業

30 他の会社または企業

Q13　新型コロナウイルスに関する情報源として、以下の情報源から十分に情報発信が行われていると思いますか？それぞれ4段階でお選びください。（SA）

1 医師
2 看護師
3 薬剤師
4 獣医
5 歯科医
6 健康フェア・イベント
7 新聞
8 雑誌
9 本
10 科学文献（論文）
11 テレビ
12 ラジオ
13 ネットのニュースサイト
14 検索エンジン（Google、Yahooなど）
15 LINE／ライン
16 Facebook／フェイスブック
17 Twitter／ツイッター
18 Instagram／インスタグラム
19 YouTube／ユーチューブ
20 TikTok／ティックトック
21 医療系情報サイト（Medical Note、MEDLEY 、アピタルなど）
22 ブログまたは有名人、著名人のウェブページ
23 都道府県や市町村などの自治体
24 政府
25 新型コロナウイルス感染症対策専門家会議（現分科会）
26 友達
27 家族
28 科学者や研究者
29 製薬企業
30 他の会社または企業

Q14　新型コロナウイルスのワクチン接種の判断に、以下の情報源から情報はどの程度参考にしましたか？それぞれ4段階でお選びください。（SA）

1 医師
2 看護師
3 薬剤師

4　獣医
5　歯科医
6　健康フェア・イベント
7　新聞
8　雑誌
9　本
10　科学文献（論文）
11　テレビ
12　ラジオ
13　ネットのニュースサイト
14　検索エンジン（Google、Yahooなど）
15　LINE／ライン
16　Facebook／フェイスブック
17　Twitter／ツイッター
18　Instagram／インスタグラム
19　YouTube／ユーチューブ
20　TikTok／ティックトック
21　医療系情報サイト（Medical Note、MEDLEY 、アピタルなど）
22　ブログまたは有名人、著名人のウェブページ
23　都道府県や市町村などの自治体
24　政府
25　新型コロナウイルス感染症対策専門家会議（現分科会）
26　友達
27　家族
28　科学者や研究者
29　製薬企業
30　他の会社または企業

Q15　新型コロナウイルスの感染を懸念して、医療機関等の受診を控えた経験はありますか？（MA）

1　定期通院を控えたことがある
2　風邪症状や頭痛、腹痛などの突発的症状による受診を控えたことがある
3　健康診断や人間ドッグ、がん検診を控えた
4　その他の受診を控えた
5　いいえ

Q16　新型コロナウイルスのワクチンを1回でも接種しましたか？（SA）

1　市区町村が実施する「集団接種」あるいは「個別接種」で1回目を受けた（接種予約済み含む）
2　国や都道府県が実施する「大規模接種」で1回目を受けた（接種予約済み含む）

3　企業や大学の実施する「職域接種」で1回目を受けた（接種予約済み含む）
4　受けたいがまだ接種予約ができていない
5　受けるかどうか迷っている
6　受けるつもりはない
7　関心がない

Q17　既にワクチン接種を受けた方は1回目を受けた当時、受けていない方は今現在、ご自身の周りではどれくらいの方が接種を受けていますでしょうか（いましたでしょうか）。あなたの感覚に最も近いものをお答えください。（SA）

1　0%
2　25%
3　50%
4　75%
5　100%

Q18　新型コロナウイルスのワクチン接種を受けた（接種予約した）のはどのような理由がありますか？（MA）

■　新型コロナウイルスのワクチンに関する特定の懸念
1　短期的な副反応・安全性への懸念がある程度払拭された
2　短期的な副反応・安全性への懸念よりワクチン接種のメリットが上回った
3　将来の副反応・安全性への懸念がある程度払拭された
4　将来の副反応・安全性への懸念よりワクチン接種のメリットが上回った
5　有効性への懸念がある程度払拭された
6　有効性への懸念よりワクチン接種のメリットが上回った
7　その他（自由記述）
■　新型コロナウイルスのワクチンに限らず、ワクチン接種全般への姿勢が変わった
8　ワクチンは必要と思いはじめた（病気にかかるリスクはない等）
9　ワクチンが有効だと思いはじめた
10　ワクチンの副反応・安全性への懸念がある程度払拭された
11　ワクチンに対しての恐怖感がある程度払拭された
12　その他（自由記述）

■　新型コロナウイルスのワクチン接種を判断するのに必要な情報が増えた
13　個人の健康状態との適合性（例えば、アレルギー、併存疾患）
14　身近な人が受けているかどうか
15　その他（自由記述）

■　新型コロナウイルスのワクチンに関係する組織・個人への信頼が高くなった
16　公的機関や政治家に対して
17　科学者に対して

18　製薬会社に対して
19　医療関係者に対して
20　その他（自由記述）

■　その他
21　家族が新型コロナウイルスに感染したから
22　友人や職場の同僚が新型コロナウイルスに感染したから
23　家族が接種を受けた・予約したから
24　友人や職場の同僚が接種を受けた・予約したから
25　職場の同僚が接種を受けた・予約したから
26　主治医やかかりつけ医に勧められたから
27　仕事や人間関係をスムーズに進めるため
28　タレントや著名人に影響を受けた
29　ワクチンの接種会場や接種できる医療機関が近くにある
30　新型コロナウイルスの空気感染が心配
31　緊急事態宣言やまん延防止等重点措置による外出抑制効果の薄れが心配
32　医療の逼迫が心配
33　感染状況が心配
34　デルタ株などの変異株の蔓延が心配
35　ワクチン接種はパンデミック収束には欠かせない
36　その他（自由記述）

Q19　新型コロナウイルスのワクチン接種を受けたくない理由、あるいはワクチンを接種するかどうかわからないのは、どんな理由がありますか？（MA）

■　新型コロナウイルスのワクチンに関する特定の懸念
1　短期的な副反応・安全性
2　将来の副反応・安全性
3　有効性
4　ワクチン瓶内の異物や不純物
5　その他（自由記述）

■　新型コロナウイルスのワクチンに限らず、ワクチン接種全般への姿勢
6　ワクチンは必要ない（病気にかかるリスクはない等）
7　ワクチンが有効だとは思わない
8　ワクチンの副反応・安全性に懸念がある
9　ワクチンに対して恐怖感がある
10　その他（自由記述）

■　新型コロナウイルスのワクチン接種を判断するにはもっと情報が必要

11　個人の健康状態との適合性（例えば、アレルギー、併存疾患）
12　身近な人が受けているかどうか
13　その他（自由記述）

■　新型コロナウイルスのワクチンに関係する組織・個人への信頼
14　公的機関や政治家に対して
15　科学者に対して
16　製薬会社に対して
17　医療関係者に対して
18　その他（自由記述）

■　その他
19　リスクの高い人（高齢者や基礎疾患ある人）が最初にワクチンを受けることを望む
20　若い人が最初にワクチンを受けることを望む
21　ワクチン接種のために医療機関へ行くのが怖い
22　接種後の副反応による仕事や生活への支障が、ワクチン接種のメリットを上回る
23　変異種にはワクチンの効果があまり期待できない
24　ワクチンの接種会場や接種できる医療機関が近くにない
25　ワクチン接種やパンデミック収束にはあまり関係ない
26　その他（自由記述）

Q20　新型コロナウイルスのワクチンについて、メリットはどのくらい大きいと感じていますか？あなたの認識でお答えください。（SA）

1　とても小さい
2　小さい
3　どちらでもない
4　大きい
5　とても大きい

Q21　新型コロナウイルスのワクチンについて、デメリットはどのくらい大きいと感じていますか？あなたの認識でお答えください。（SA）

1　とても小さい
2　小さい
3　どちらでもない
4　大きい
5　とても大きい

Q22　他の人が新型コロナウイルスのワクチンを接種していたら、自分もワクチン接種をするべきだと思いますか？（SA）

1　まったくそう思わない
2　そう思わない
3　どちらでもない
4　そう思う
5　強くそう思う

Q23　新型コロナウイルスのワクチンを接種したら、不安が和らぐと思いますか？（SA）

1　まったくそう思わない
2　そう思わない
3　どちらでもない
4　そう思う
5　強くそう思う

Q24　新型コロナウイルスのワクチンの開発分野における科学者を信頼していますか？（SA）

1　まったく信頼していない
2　信頼していない
3　どちらでもない
4　信頼している
5　強く信頼している

Q25　新型コロナウイルスのワクチンの承認について、公的機関を信頼していますか？（SA）

1　まったく信頼していない
2　信頼していない
3　どちらでもない
4　信頼している
5　強く信頼している

Q26　新型コロナウイルスのワクチン接種について、医療関係者を信頼していますか？（SA）

1　まったく信頼していない
2　信頼していない
3　どちらでもない
4　信頼している
5　強く信頼している

Q27　どういったお仕事内容の方が、積極的にワクチン接種をすべきだと思いますか？（MA）

1　オフィスワーク中心（事務・企画・開発など）
2　外回り中心（営業など）
3　オフィス以外の職場での仕事中心（生産・製造・現場など）
4　接待を伴わない飲食提供
5　接待を伴う飲食提供
6　教育
7　医療
8　ヘルパー・介護
9　タクシードライバー
10　運送
11　小売り（店舗含む）
12　宿泊業・レジャー関連
13　保育
14　理容・美容・エステ
15　官公庁（国と地方公共団体の役所）
16　その他、収入のある仕事をしている
17　学生
18　専業主ふ
19　その他
20　特にない

Q28　新型コロナウイルスのワクチン接種を終了した人としていない人（あるいは各種検査での陰性証明の有無）で、様々な活動制限が変わることをどう思いますか？（SA）

1　賛成
2　反対
3　どちらとも言えない

Q29　新型コロナウイルスのワクチン接種を終了した人としていない人（あるいは各種検査での陰性証明の有無）で、様々な活動制限が変わる場合、あなたは接種しますか？（SA）

1　はい
2　いいえ
3　わからない

Q30　新型コロナウイルス感染症のワクチンについて、今後、12歳未満の子どもも接種できるようになった場合、あなたは次のどれにあてはまりますか？（SA）

1　賛成（該当する年齢の子どもがいる）

2　賛成（該当する年齢の子どもはいない）

3　反対（該当する年齢の子どもがいる）

4　反対（該当する年齢の子どもはいない）

5　どちらとも言えない（該当する年齢の子どもがいる）

6　どちらとも言えない（該当する年齢の子どもはいない）

Q31　３回目の新型コロナウイルスのワクチン接種が行えるようになったら、接種しますか？（SA）

1　はい

2　いいえ

3　わからない

4　既に接種した

Questionnaire translated into English for this report

Q1 In Internet-based surveys, the problem is that some people lie or do not read the questions and give lame answers. Will you read the questions and answer them seriously? If you are willing to answer seriously, please check the box below and proceed to the next page. (SA)

1 I will read the questions answer them seriously
2 Decline to answer

Q2 Which prefecture do you live in? (SA)

1 Hokkaido Prefecture
2 Aomori Prefecture
3 Iwate Prefecture
4 Miyagi Prefecture
5 Akita Prefecture
6 Yamagata Prefecture
7 Fukushima Prefecture
8 Ibaraki Prefecture
9 Tochigi Pref.
10 Gunma Prefecture
11 Saitama Prefecture
12 Chiba Prefecture
13 Tokyo Prefecture
14 Kanagawa Prefecture
15 Niigata Prefecture
16 Toyama Prefecture
17 Ishikawa Prefecture
18 Fukui Prefecture
19 Yamanashi Prefecture
20 Nagano Prefecture
21 Gifu Prefecture
22 Shizuoka Prefecture
23 Aichi Prefecture
24 Mie Prefecture
25 Shiga Prefecture
26 Kyoto Prefecture
27 Osaka Prefecture
28 Hyogo Prefecture
29 Nara Prefecture

30 Wakayama Prefecture
31 Tottori Prefecture
32 Shimane Prefecture
33 Okayama Prefecture
34 Hiroshima Prefecture
35 Yamaguchi Prefecture
36 Tokushima Prefecture
37 Kagawa Prefecture
38 Ehime Prefecture
39 Kochi Prefecture
40 Fukuoka Prefecture
41 Saga Prefecture
42 Nagasaki Prefecture
43 Kumamoto Prefecture
44 Oita Prefecture
45 Miyazaki Prefecture
46 Kagoshima Prefecture
47 Okinawa Prefecture

Q3 How is your current health condition? (SA)

1 Very good
2 Good
3 Fair
4 Poor
5 Very poor

Q4 How confident are you when filling out medical forms by yourself? (SA)

1 Not at all
2 A little bit
3 Somewhat
4 Fairly
5 Extremely

Q5 Have you ever received a COVID-19 test? (MA)

1 No
2 Yes, PCR test
3 Yes, antigen test

	4	Yes, antibody test
	5	Yes, unsure what kind.

Q6 How anxious are you about COVID-19? (SA)

1	Not at all anxious
2	Vaguely anxious
3	Have a clear sense of anxiety
4	Feel fear and have anxiety

Q7 To what extent did the COVID-19 pandemic affect your life within the past year? (SA)

1	Not at all
2	Not much
3	Somewhat
4	Quite a lot

Q8 Do you engage in preventive measures against COVID-19? (MA)

1	Avoid places with poor ventilation
2	Avoid densely packed places
3	Avoid speaking when nearby others
4	Wash hands, gargle, and/or sanitize hands with alcohol
5	Cover mouth when coughing/sneezing
6	Telework
7	Social distancing
8	Wear a mask when out
9	Avoid dinners/parties with many guests
10	Avoiding meals with non-household members
11	Ventilation
12	Minimize outings
13	Other
14	None in particular

Q9 What is your best guess as to whether you will get COVID-19 within the next 6 months? (SA)

1	I don't think I will get COVID-19
2	I think I will get a mild case of COVID-19
3	I think I will get seriously ill from COVID-19
4	I have already had COVID-19

Q10 Have any family, friends, or colleagues been infected with COVID-19? (MA)

1 Family (mild illness)
2 Family (moderate illness)
3 Family (severe illness)
4 Family (unsure of severity)
5 Friends and/or colleagues (mild illness)
6 Friends and/or colleagues (moderate illness)
7 Friends and/or colleagues (severe illness)
8 Friends and/or colleagues (unsure of severity)
9 None/Unsure

Q11 From what sources do you receive information about COVID-19? (MA)

1 Physicians
2 Nurses
3 Pharmacists
4 Veterinarians
5 Dentists
6 Health fairs & events
7 Newspapers
8 Magazines
9 Books .
10 Scientific literature
11 Television
12 Radio
13 Internet news sites
14 Search engines (Google, Yahoo, etc.)
15 LINE
16 Facebook
17 Twitter
18 Instagram
19 YouTube
20 TikTok
21 Medical information sites
22 Blogs or celebrity web pages
23 Local authorities such as prefectures and municipalities
24 Government
25 Medical task forces

26 Friends
27 Familiy members
28 Scientists and researchers
29 Pharmaceutical companies
30 Other companies

Q12 How much do you trust information about COVID-19 from the following sources? (4-point scale) (SA)

1 Physicians
2 Nurses
3 Pharmacists
4 Veterinarians
5 Dentists
6 Health fairs & events
7 Newspapers
8 Magazines
9 Books .
10 Scientific literature
11 Television
12 Radio
13 Internet news sites
14 Search engines (Google, Yahoo, etc.)
15 LINE
16 Facebook
17 Twitter
18 Instagram
19 YouTube
20 TikTok
21 Medical information sites
22 Blogs or celebrity web pages
23 Local authorities such as prefectures and municipalities
24 Government
25 Medical task forces
26 Friends
27 Familiy members
28 Scientists and researchers
29 Pharmaceutical companies
30 Other companies

Q13 Do you believe the following COVID-19 information sources are sufficiently disseminated? (4-point scale) (SA)

1 Physicians
2 Nurses
3 Pharmacists
4 Veterinarians
5 Dentists
6 Health fairs & events
7 Newspapers
8 Magazines
9 Books .
10 Scientific literature
11 Television
12 Radio
13 Internet news sites
14 Search engines (Google, Yahoo, etc.)
15 LINE
16 Facebook
17 Twitter
18 Instagram
19 YouTube
20 TikTok
21 Medical information sites
22 Blogs or celebrity web pages
23 Local authorities such as prefectures and municipalities
24 Government
25 Medical task forces
26 Friends
27 Familiy members
28 Scientists and researchers
29 Pharmaceutical companies
30 Other companies

Q14 To what extent did you consult information from the following sources in making your decision to vaccinate against COVID-19? (4-point scale) (SA)

1 Physicians
2 Nurses
3 Pharmacists
4 Veterinarians
5 Dentists

6 Health fairs & events
7 Newspapers
8 Magazines
9 Books .
10 Scientific literature
11 Television
12 Radio
13 Internet news sites
14 Search engines (Google, Yahoo, etc.)
15 LINE
16 Facebook
17 Twitter
18 Instagram
19 YouTube
20 TikTok
21 Medical information sites
22 Blogs or celebrity web pages
23 Local authorities such as prefectures and municipalities
24 Government
25 Medical task forces
26 Friends
27 Familiy members
28 Scientists and researchers
29 Pharmaceutical companies
30 Other companies

Q15 Have you ever refrained from visiting a medical institution because you were anxious about being infected by the new coronavirus? (MA)

1 Yes, for regular visits
2 Yes, for sudden symptoms such as cold, headache, abdominal pain, etc.
3 Yes, for routine physicals and/or cancer screening
4 Yes, for other appointments
5 No

Q16 Have you received at least one dose of vaccine for new coronavirus or scheduled for vaccination? (SA)

1 Yes, "mass vaccination" or "individual vaccination" administered by municipalities
2 Yes, "mass vaccination" administered by the national or prefectural government
3 Yes, "workplace vaccination" administered by a company or university

4 Have not made a reservation but intend to be vaccinated
5 Unsure regarding vaccination
6 Do not intend to be vaccinated
7 Do not have any interests

Q17 If you have already been vaccinated, how many people around you were vaccinated at the time you received the first dose, and if not, how many people around you are currently vaccinated? (SA)

1 About 0%
2 About 25%
3 About 50%
4 About 75%
5 About 100%

Q18 What is the reason why you were vaccinated (made an appointment for vaccination) against the new coronavirus? (MA)

■ Dispelled specific concerns about the COVID-19 vaccine
1 Concerns about short-term adverse reactions and safety have been dispelled to some extent
2 Benefits of vaccination outweigh concerns about short-term adverse reactions and safety
3 Concerns about future adverse reactions and safety have been dispelled to some extent
4 Benefits of vaccination outweigh concerns about future adverse reactions and safety
5 Concerns about effectiveness have been dispelled to some extent
6 Benefits of vaccination outweigh concerns about effectiveness
7 Others (free answer)

■ Change in attitude toward vaccination in general, not just the COVID-19 vaccine
8 I have begun to believe that vaccines are necessary
9 I have begun to believe that vaccines are effective
10 Concerns about adverse reactions and the safety of vaccines have been dispelled to some extent
11 Fear of vaccines has been dispelled to some extent
12 Others (free answer)

■ Availability of the information necessary to make a decision about COVID-19 vaccination
13 There is now more information about the compatibility of vaccines with certain pre-existing health conditions (e.g., allergies)
14 There is now more information regarding whether or not someone close to oneself has received the vaccine.
15 Others (free answer)

■ Increased trust in organizations and individuals involved with the COVID-19 vaccine
16 Trust in public institutions and politicians has increased

17 Trust in scientists has increased
18 Trust in pharmaceutical companies has increased
19 Trust in healthcare professionals has increased.
20 Others (free answer)

■ Others

21 A family member has been infected with COVID-19
22 A friend or colleague at work has been infected with COVID-19
23 A family member has received COVID-19 vaccination
24 A friend has received COVID-19 vaccination
25 A colleague at work has received COVID-19 vaccination
26 My doctor or family doctor recommended COVID-19 vaccination
27 My work and personal relationships would improve if I received the COVID-19 vaccination
28 I have been influenced by a TV personality or celebrity
29 There is a vaccination site or medical facility near my home where I can receive the vaccine
30 I was concerned about airborne transmission of COVID-19
31 I was worried about the diminishing effect of the emergency declarations and other COVID-19 measures to prevent the spread of the virus
32 I worried about the overwhelmed healthcare system
33 I worried about the infection situation
34 I worried about the spread of mutant strains such as the Delta variant
35 I was beginning to think that vaccination is indispensable to contain the pandemic
36 Others (free answer)

Q19 What is the reason why you were unsure or unwilling to take a vaccine against the new coronavirus? (MA)

■ Specific anxiety about the COVID-19 vaccine

1 Short-term adverse reactions and safety
2 Future adverse reactions and safety
3 Effectiveness
4 Foreign matter or impurities in the vaccine bottle
5 Others (free answer)

■ Attitude toward vaccination in general, not limited to the new coronavirus vaccine

6 No vaccines are required (no risk of contracting the disease, etc.)
7 I don't believe the vaccine is effective
8 Concerns about adverse reactions and safety of vaccines
9 Fear of vaccines
10 Others (free answer)

■ More information is needed to decide whether or not to get a COVID-19 vaccine

11 Compatibility with personal health conditions (e.g., allergies, comorbidities)

12 Whether other people have been vaccinated or not

13 Others (free answer)

■ Lack of trust

14 In public institutions and politicians has increased

15 In scientists has increased

16 In pharmaceutical companies has increased

17 In healthcare professionals has increased.

18 Others (free answer)

■ Others

19 I want those at higher risk (elderly and those with underlying medical conditions) to be the first to receive the vaccine

20 I want young people to be the first to receive the vaccine

21 I am afraid to go to a medical institution for vaccination

22 The disruption to work and life due to adverse reactions after vaccination outweighs the benefits of vaccination

23 I do not expect vaccines to be very effective against mutant species

24 Vaccine sites and medical facilities are not located close to my home

25 Vaccination has little to do with pandemic containment

26 Others (free answer)

Q20 How do you feel are the benefits of the COVID-19 vaccine? (SA)

1 Very small

2 Small

3 Medium

4 Large

5 Very large

Q21 How do you think the disadvantages of the COVID-19 vaccine are? (SA)

1 Very small

2 Small

3 Medium

4 Large

5 Very large

Q22 If others have been vaccinated against COVID-19, I believe I should be vaccinated as well. (SA)

1	Strongly disagree
2	Disagree
3	Neither agree nor disagree
4	Agree
5	Strongly agree

Q23 Getting the COVID-19 vaccine will ease my anxiety. (SA)

1	Strongly disagree
2	Disagree
3	Neither agree nor disagree
4	Agree
5	Strongly agree

Q24 Do you trust scientists in the field of vaccine development for COVID-19? (5-point scale) (SA)

Q25 Do you trust the public authorities who approve vaccines for COVID-19? (5-point scale) (SA)

Q26 Do you trust your healthcare provider about vaccination against COVID-19? (5-point scale) (SA)

Q27 For which professions do you believe vaccination should be prioritized? (MA)

1	Office workers (e.g., clerical, planning, development, etc.)
2	Non-office workers (e.g., sales)
3	Non-office workers (e.g., production, manufacturing, on-site, etc.)
4	Food and beverage provision without entertainment
5	Food and beverage provision with entertainment
6	Education
7	Medical care
8	Nursing and homecare
9	Cab drivers
10	Transportation
11	Retail
12	Lodging and leisure
13	Childcare
14	Hairdessing, beauty, and aesthetics
15	Government offices
16	Other income-earning jobs

17 Students
18 Homemakers
19 Other
20 None in particular

Q28 Do you support or oppose changing various activity restrictions depending on vaccination status (or whether or not one has proof of negative testing)? (SA)

1 Support.
2 Oppose
3 neither support nor oppose

Q29 Do you receive a vaccine if various activity restrictions change depending on vaccination status (or whether or not one has proof of negative testing)? (SA)

1 Yes
2 No
3 Not sure

Q30 Which of the following would apply to you if the COVID-19 vaccination were made available to children under 12 years of age in the future? (SA)

1 For (have children in the specified age range)
2 For (do not have children in the specified age range)
3 Against (have children in the specified age range)
4 Against (do not have children in the specified age range)
5 Neither for nor against (have children in the specified age range)
6 Neither for nor against (do not have children in the specified age range)

Q31 When the third dose becomes available, will be vaccinated? (SA)

1 Yes
2 No
3 Not sure
4 Already vaccinated
